# Genomic diversification, adaptive convergence, and regulatory rewiring in aging *Escherichia coli* colonies

**DOI:** 10.1186/s12866-026-04983-z

**Published:** 2026-03-31

**Authors:** Claude Saint-Ruf, Adrien Launay, Olivier Tenaillon, Ivan Matic

**Affiliations:** 1 Université Paris Cité, Inserm U1016, CNRS UMR 8104, Institut Cochin, Paris, F-75014 France; 2 Endogenomiks S.A. de C.V., Guadalajara, Mexico

**Keywords:** *Escherichia coli*, Structured microbial populations, Regulatory rewiring, Adaptive evolution, Nutrient limitation, *cspC-yobF* operon, Genomic diversification

## Abstract

**Background:**

Bacterial colonies are dynamic evolutionary microenvironments where spatial heterogeneity and nutrient gradients generate diverse ecological niches. Understanding how such structured environments drive genetic and functional diversification, including the emergence of clinically relevant traits such as antibiotic resistance, remains a major challenge.

**Methods:**

To dissect adaptive strategies in structured populations, we performed whole-genome sequencing on 24 *Escherichia coli* isolates recovered from a three-week-old colony. Transcriptomic profiling was conducted on two representative mutants, and targeted competition assays were used to assess fitness relative to the parental strain.

**Results:**

Genome sequencing uncovered extensive evolutionary diversification, with 34 distinct mutations, mostly insertion-sequence events, affecting transcriptional, stress-response, metabolic, and envelope regulators. Strikingly, half of all isolates carried mutations in the *yobF-cspC* operon, identifying it as a major adaptive hotspot in structured populations. Transcriptomic analyses revealed a broad regulatory shift in *yobF-cspC* mutants, with substantial activation of central metabolism and biosynthesis coupled to repression of acid resistance and stress pathways. Functionally, deletion of *cspC* alone conferred a robust σS-independent fitness advantage and fully restored competitiveness in an *rpoS*-inactivated background in aging colonies.

Beyond this dominant adaptive trajectory, genome analysis revealed isolates carrying mutations that confer gain-of-function phenotypes, including β-lactam and rifamycin resistance, despite the absence of antibiotic pressure, highlighting aging colonies as potential reservoirs of clinically relevant diversity.

**Conclusions:**

Our results suggest that aging bacterial colonies can serve as incubators of evolutionary innovation, simultaneously generating diverse functional variants while selecting for metabolic specialization. Together, these findings show how spatial structure and nutrient recycling shape bacterial adaptation and diversification, providing a powerful and tractable model to investigate evolutionary processes in natural and pathogenic communities.

**Supplementary Information:**

The online version contains supplementary material available at 10.1186/s12866-026-04983-z.

## Introduction

Bacteria thrive in diverse ecological niches thanks to rapid growth, large population sizes, and remarkable phenotypic plasticity [1–3]. In natural environments, they predominantly inhabit structured communities, such as biofilms and colonies. Although both forms are widespread, colonies remain comparatively understudied despite their importance in natural and host-associated ecosystems [4–6]. Biofilms contain cells embedded in a robust extracellular matrix firmly anchored to a surface, which facilitates experimental procedures [7, 8]. In contrast, colonies form soft, dome-shaped multicellular assemblies lacking this dense matrix. Moreover, the fragile architecture of colonies complicates handling and limits the standardization of analytical protocols [8–10].

Recent physics-driven analyses [8, 11] have renewed interest in colonies by emphasizing their strong coupling between metabolism, diffusion, growth mechanics, and demographic noise. Consistent with this view, cytometric “biopsy” analyses revealed spatially segregated subpopulations of vegetative, dormant, and dying cells within colonies [12], underscoring their value as a model for microbial evolution in temporally and spatially heterogeneous environments.

Unlike well-mixed planktonic cultures dominated by clonal interference and rapid selective sweeps, structured populations foster diversification by imposing local competition, steep metabolic gradients, and nutrient recycling [2, 13]. Such environments promote coexistence, adaptive trade-offs, and resilience to ecological uncertainty, akin to the “insurance hypothesis” in ecology [13–15].

Clinically, *E. coli* forms surface-associated communities in catheter-associated urinary tract infections, chronic infections, and device-related biofilms that develop steep nutrient/oxygen gradients [16, 17]. Within-patient diversification has been documented in “deep and closed” visceral infections, where micro-heterogeneity emerges along the self-preservation vs. nutritional competence (SPANC) axis [18, 19].

Even the “nutrient-rich” mammalian intestine imposes spatial/temporal nutrient heterogeneity through feeding cycles and mucus microhabitats [20]. Outside hosts, *E. coli* persists in soil, water, and food surfaces where resource availability fluctuates severely [21, 22]. In these secondary niches, proliferation/starvation cycles select for stationary-phase physiology and metabolic specialization.

Agar colony aging provides a tractable, experimentally controllable model for evolutionary responses in such structured, nutrient-depleted communities. In our previous study [23], we established dedicated observational tools that enabled high-resolution monitoring of colony architecture over time and showed that aging *Escherichia coli* colonies on agar develop islands of actively growing cells embedded within layers of dormant or dying cells, often surrounding localized cores of dead cells. As waste accumulates and nutrients dwindle, surviving cells diversify phenotypically in motility, carbon utilization, and stress resistance, with several variants gaining substantial fitness [23].

Building on these findings, we observed that colonies not only promote diversification of competitive lineages but also allow persistence of functionally specialized and potentially clinically relevant variants that would likely be outcompeted in well-mixed populations.

The genetic drivers of this diversification remain poorly understood. We integrated whole-genome sequencing and phenotypic analyses of 24 evolved isolates from a three-week-old colony to characterize the genetic heterogeneity underlying colony diversification. We identified mutational events affecting transcriptional regulation, stress responses, carbon metabolism, and envelope biogenesis. Among these, recurrent mutations in the *yobF-cspC* operon [24, 25] emerged as a prominent evolutionary trajectory. *cspC*, encoding an RNA chaperone, plays a key role in the regulation of stress response and gene expression in *E. coli* [24]. Under the aging-colony conditions tested here, combining transcriptomic profiling with competitive assays, we provide evidence that *cspC* inactivation is a major hotspot of metabolic rewiring, consistent with a shift from stress resilience toward metabolic flexibility in structured colony environments.

## Results

### Phenotype and fitness

In our previous study, we provided a detailed phenotypic characterization of 25 evolved *E. coli* strains isolated from a 3-week-old colony [23]. These independent isolates, all derived from the parental pygYFP strain, were arbitrarily designated Y1 to Y25 at the time of isolation. We assessed their competitive ability against the WT strain in mixed colonies (competitive index in colony, CIC), as well as swarming motility, paraquat resistance, biofilm formation, tolerance to 33 antibiotics, and the capacity to metabolize 95 carbon sources. While each strain displayed a unique phenotypic profile, several traits recurred across independent isolates, most notably, increased swarming motility, reduced biofilm formation index (BFI), and improved acetate utilization.

**Fig. 1:**
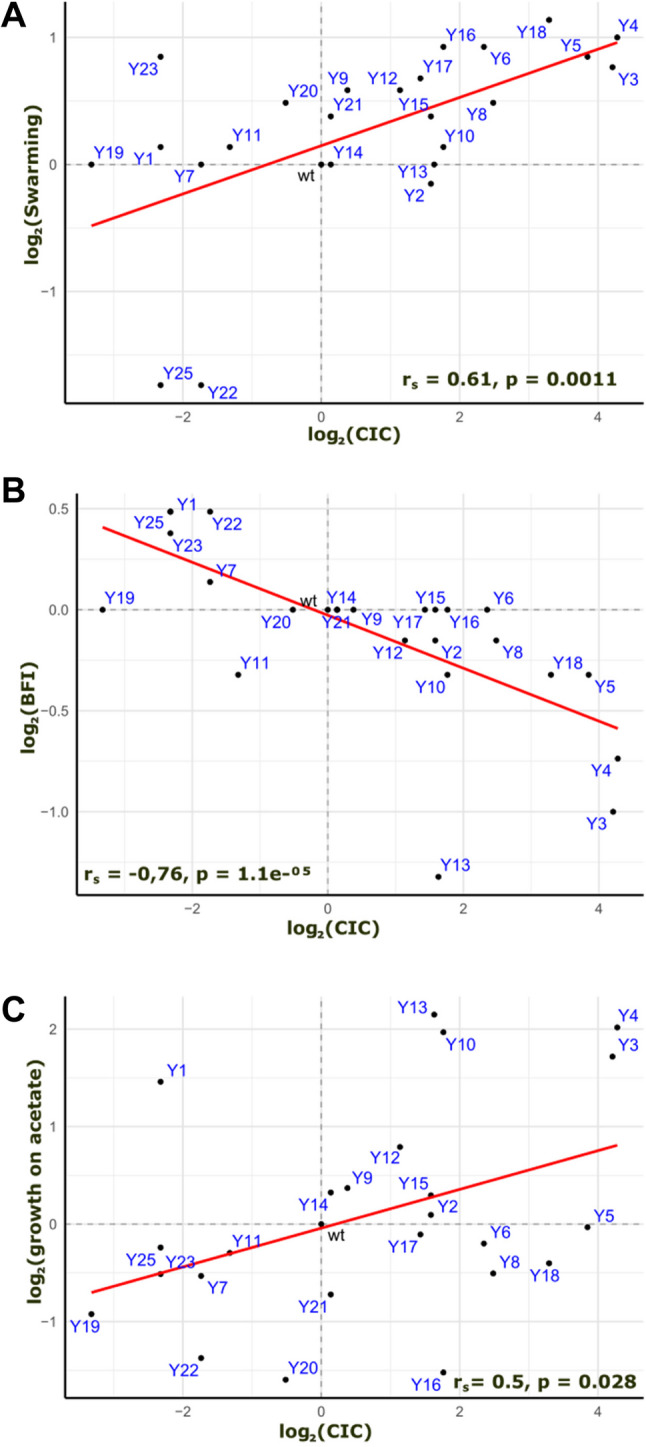
Trade-offs between motility, biofilm formation, acetate metabolism, and competitiveness. **A** Swarming motility plotted against the colony competitive index (CIC). **B** Biofilm formation index (BFI) plotted against CIC. **C** Growth on acetate plotted against CIC. Data for motility, BFI, and growth on acetate were obtained from [23]. CIC values correspond to colony competitive indices measured in mixed-colony assays against the parental strain. Spearman’s rank correlation coefficients (rₛ) and *p*-values are indicated on each panel

Upon reanalyzing the phenotypic data from 24 of the 25 isolates, Spearman’s rank correlation analyses (two-sided tests) revealed that CIC positively correlated with both swarming (rₛ = 0.614, *p* = 0.0011) and growth on acetate (rₛ = 0.472, *p* = 0.028), whereas BFI negatively correlated with CIC (rₛ = −0.544, *p* = 0.007) (Fig. 1). Swarming and BFI were themselves negatively correlated (rₛ = − 0.76, *p* = 1.1 × 10⁻⁵), consistent with the antagonistic regulation of these behaviors in *E. coli* [26]. Low biofilm formation and enhanced swarming are commonly associated with a physiological shift toward metabolic activity and away from stationary-phase stress responses. Thus, the observed link between acetate assimilation and competitive fitness may reflect adaptive reprogramming under conditions of resource limitation.

We therefore sequenced the whole genomes of the 24 isolates to identify mutations that might underlie the emergence of these phenotypes.

### Genome-wide mutagenesis in aged colonies

Whole-genome sequencing of the 24 isolates was performed using Illumina technology. Breseq analysis [27] revealed between one and three mutations per strain, resulting in 34 distinct mutational events across 22 isolates affecting 66 genes (supplementary Figure S1A). The distribution of mutations is summarized in Table 1 and Supplementary Table S1.

**Table 1:**
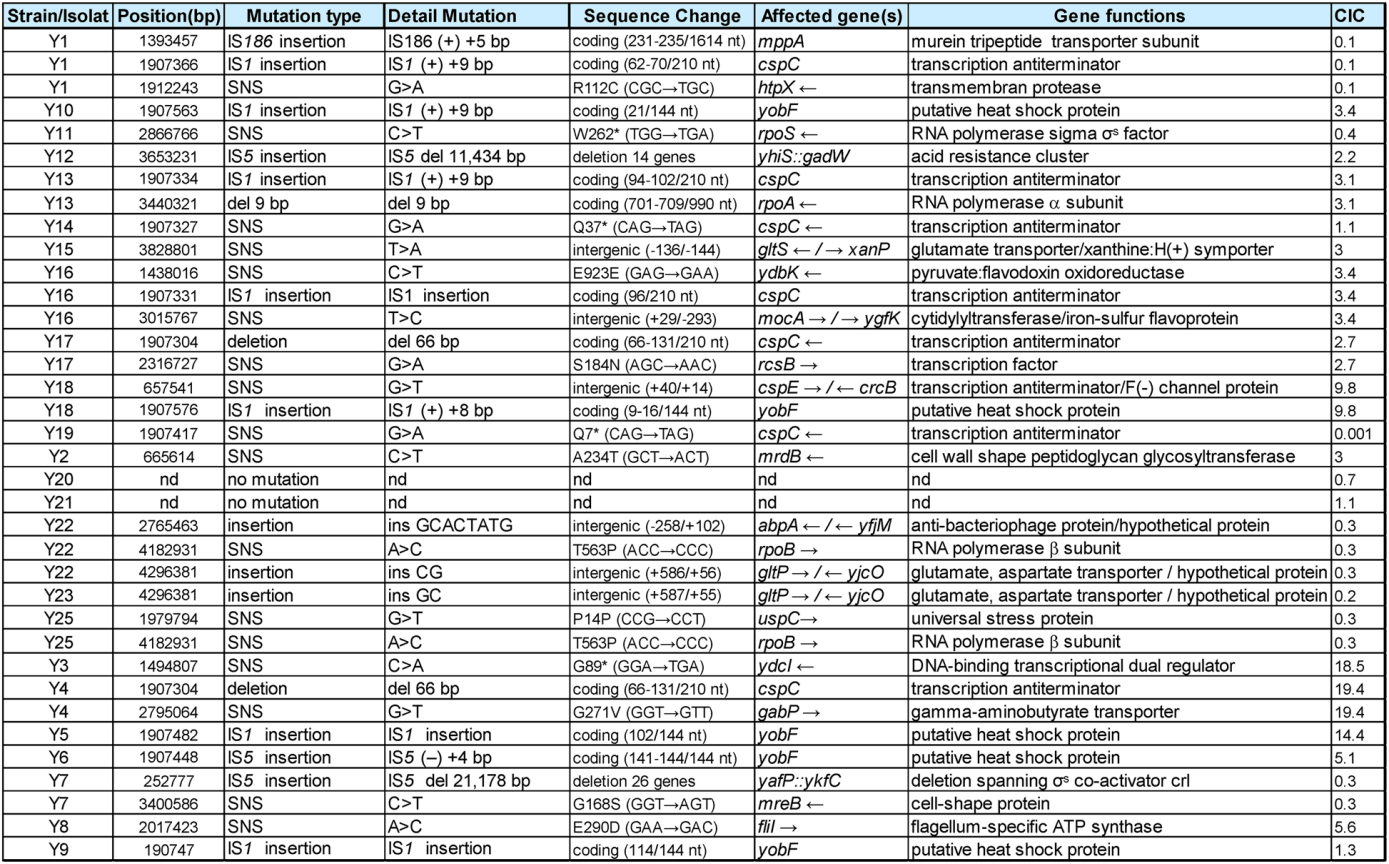
Mutations identified in 24 *E. coli* isolates from a 3-week-old colony. Whole-genome sequencing of evolved strains was performed using Illumina technology and analyzed with *breseq* [27]. For each isolate, the table lists the genomic position, mutation type, sequence change, affected gene(s), and annotated function. CIC values correspond to the colony competitive index measured in mixed-colony assays against the wild-type strain [23]. IS insertions are annotated by element and orientation relative to the reference genome. No mutation was detected in isolates Y20 and Y21.

Thirteen mutations were mediated by insertion sequences (IS), including seven IS*1*, three IS*5*, and one IS*186*/IS*421*, found across 12 strains. This observation is consistent with our previous transcriptomic data [23], which showed increased expression of IS-encoded transposases in aging colonies, particularly at day 7. Together, these data suggest that IS mobilization plays a major role in genome remodeling during colony aging, acting as a driver of diversification [28, 29] (Supplementary Figure S1A).

Two large IS-mediated deletions encompassing 26 genes (isolate Y7) and 14 genes (isolate Y12) were also detected. In addition, four short indels (three intergenic, one intragenic) and 17 single-nucleotide substitutions (SNS) were identified. Among the 14 SNS located in coding regions, four were nonsense mutations, eight were missense mutations, and two were synonymous mutations. Three SNS were located in intergenic regions.

We estimated the ratio of non-synonymous to synonymous mutations (Ka/Ks) using three approaches, including two that account for mutation biases specific to the physiological conditions of aging colonies. The simplest uncorrected estimate, based solely on raw counts (14 non-synonymous vs. 2 synonymous mutations), yielded a crude ratio of 7.0. When correcting for base substitution biases, Ka/Ks was reduced to 1.63–2.54, depending on whether the correction was based on synonymous or non-synonymous sites (see Methods). All estimates remained above the neutral expectation of 1, indicating that amino acid-altering mutations were not selectively neutral and likely conferred a selective advantage in aging colonies [30].

Taken together, these findings indicate that although mutations arise stochastically, their fixation in aging colonies is shaped by selective pressures rather than neutral drift. This suggests that the observed mutations likely contribute to adaptive diversification, prompting us to investigate their functional distribution and determine how they may influence colony-level fitness.

### Mutated genes: functional categories and impact on adaptation

**Fig. 2:**
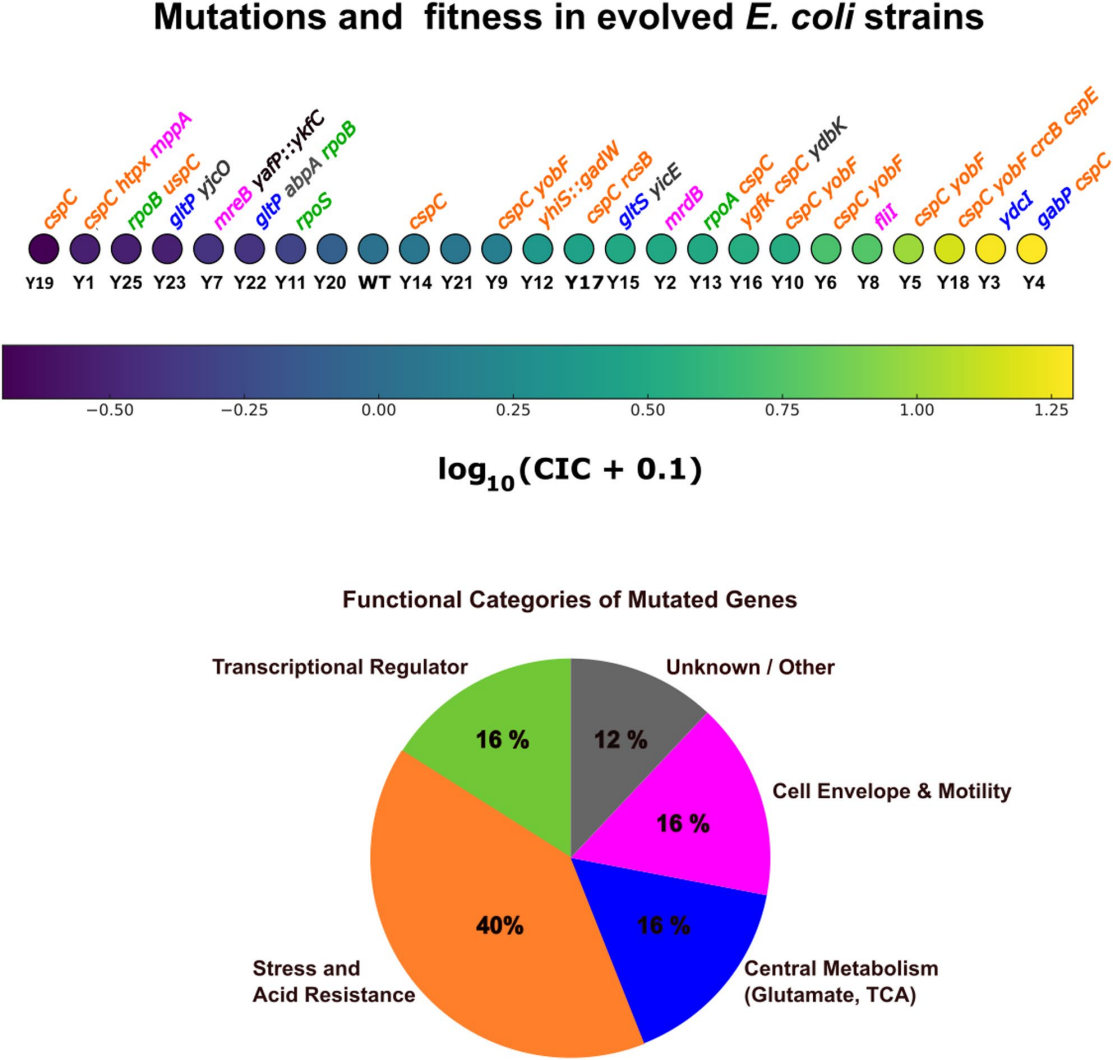
Mutational landscape and functional classification of evolved *E. coli* isolates. Distribution of mutations among the 24 evolved isolates, ordered by their colony competitive index (CIC; log₁₀ scale). The wild-type (WT) reference strain is shown for comparison. Each circle represents an isolate, and mutated genes are indicated above, color-coded according to their functional category. The pie chart below summarizes the functional distribution of all identified mutations, using the same color key. Percentages indicate the proportion of total mutations within each category

Mutations were predominantly found in genes involved in transcriptional regulation, stress adaptation, and cell envelope homeostasis (Fig. 2). To assess the biological significance of these alterations, functional enrichment analyses were performed using ShinyGO [31] and STRING [32].

The mutated genes were significantly enriched in acid and envelope stress responses (e.g., response to acidic pH, FDR = 8.7 × 10⁻⁷; envelope stress response, FDR = 0.014), in H-NS target genes (RegulonDB, FDR = 8.5 × 10⁻⁸), and in transcription-related processes such as antitermination (FDR = 0.003) and DNA-templated initiation (FDR = 0.009) (see Supplementary Figure S1B-C, Supplementary Table S2 for full details).

Interestingly, 14 of the mutated genes were previously found to be upregulated in aging wild-type (WT) colonies relative to exponential-phase cells [23]. Eight of these are directly or indirectly controlled by the general stress sigma factor RpoS (σˢ) [33, 34] (Supplementary Table S1). This overlap suggests that selection in aging colonies frequently acts on components of the σˢ-dependent network, potentially reshaping stress-response hierarchies and their regulatory links with metabolism.

Among recurrently affected genes, *rpoB* and *gltP* were each mutated in two independent isolates, while the *yobF-cspC* operon emerged as the most frequent mutational hotspot, being altered in 12 strains. Together, these findings suggest that adaptive evolution in aging colonies mainly occurs through regulatory rewiring of stress and transcription networks, particularly those involving RpoS and CspC [35–38]. Such rewiring may help optimize the balance between protection and metabolic activity in nutrient-limited, structured environments.

#### Mutations impacting the transcription machinery and stress response pathways

Whole-genome sequencing revealed mutations affecting several regulators of transcription and stress responses. However, one major recurrent trajectory, disruption of the *yobF-cspC* operon, was repeatedly associated with increased competitiveness in aging colonies.

Mutations in *rpoS or rpoB* did not improve fitness in our conditions. The *rpoS* nonsense mutant (Y11) showed no benefit despite the well-known advantages of *rpoS* loss in planktonic long-term starvation [39, 40]. Likewise, the *rpoB* T563P substitution, previously associated with rifamycin resistance and slower RNAP elongation [41, 42], did not increase competitiveness in strains Y22 and Y25. Likewise, the large IS*5*-mediated deletion in Y7, spanning *crl*, a co-activator that promotes σ^S^ binding to RNAP [43, 44], also failed to enhance fitness, consistent with *crl* being annotated as interrupted in MG1655 (EcoCyc). Together, these results suggest that broad perturbations of the transcription machinery and σ^S^-associated regulation do not, by themselves, improve competitiveness in our colony competition assays under the tested conditions.

**Fig. 3:**
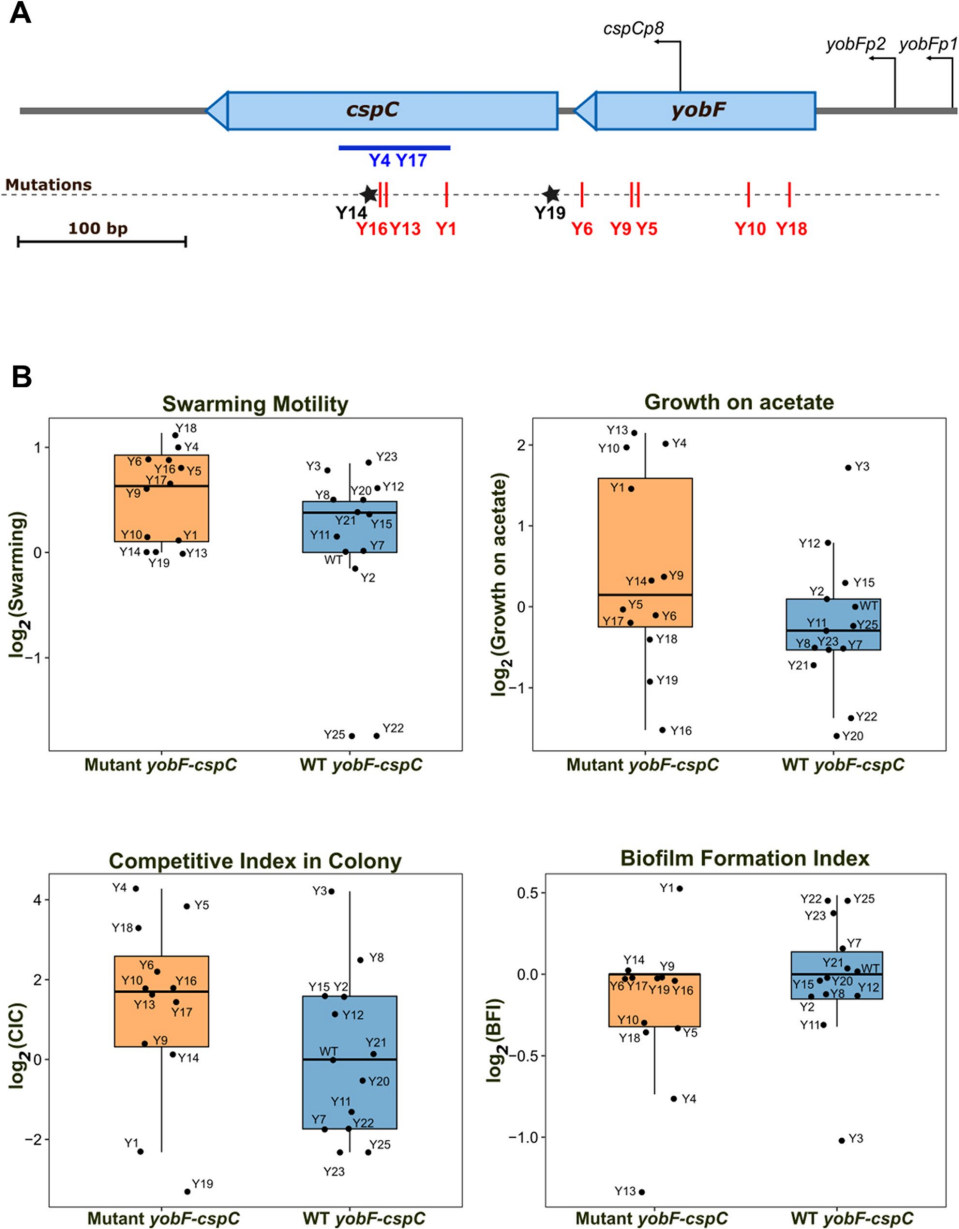
Genetic alterations in the *yobF-cspC* locus and associated traits. **A** Schematic of the *yobF-cspC* operon showing mutations detected in evolved *E. coli *isolates. The coding regions of *cspC* (210 bp) and *yobF* (144 bp) are drawn to scale and oriented by transcription direction. Red bars indicate IS insertions (IS*1*/IS*5*), black stars mark nonsense mutations introducing premature stop codons, and the blue bar denotes the 66-bp deletion observed in isolates Y17 and Y4. Mutations are mapped relative to operon structure and promoters (*yobF*p1, *yobF*p2, *cspC*p8). **B** Boxplots comparing swarming motility, growth on acetate, colony competitive index (CIC), and biofilm formation index (BFI) between isolates carrying a mutation in the *yobF-cspC* locus (“Mutant”, orange) and isolates without mutations in this locus (“WT”, blue). Values are log₂-transformed

In sharp contrast, 12 independent isolates carried mutations in the *yobF-cspC* operon, including IS insertions, nonsense or missense substitutions, and a 66-bp deletion (Fig. 3A).

Most of these mutants outcompeted the parental WT strain in mixed colonies (CIC > 1 for 9/12 isolates), and several ranked among the most competitive strains (e.g., Y4, Y5, Y6, Y10, Y13, Y18). However, a few mutants (e.g., Y1 and Y19) showed reduced fitness, and others such as Y14 displayed values close to neutrality, reflecting heterogeneity among independently evolved isolates.

To formally assess whether mutations in the *yobF–cspC* locus were associated with altered phenotypes, log₂-transformed values were compared between mutant (*n* = 12) and non-mutant (*n* = 12) isolates using two-sided Welch’s t-tests. Mutants showed higher mean log₂ values for swarming motility (0.53 vs. 0.04; t = − 1.74, df = 16.3, *p* = 0.10), acetate utilization (0.43 vs. − 0.24; t = − 1.53, df = 20.3, *p* = 0.14), and CIC (1.27 vs. 0.09; t = − 1.30, df = 21.9, *p* = 0.21), but these differences did not reach statistical significance, consistent with the marked heterogeneity in competitive outcomes among independently evolved isolates. Biofilm formation showed comparable mean log₂ values between groups (− 0.21 vs. − 0.02; t = − 1.06, df = 20.7, *p* = 0.30) (Fig. 3B).

Strains Y13 and Y18, which combined *cspC-yobF* mutations with alterations in *rpoA* (encoding the α subunit of RNA polymerase [45, 46]) or in the *cspE-crcB* region, also showed improved competitiveness (Figs. 2 and 3A), reinforcing the central contribution of the CspC pathway.

Mechanistically, CspC functions as an RNA chaperone that promotes *rpoS* mRNA translation and prevents premature transcription termination [24, 35, 36]. CspE carries partially overlapping activities [38]. Disruption of these RNA-binding regulators, especially when combined with mutations in their poorly characterized partner *yobF*, which is induced under heat stress and during the stationary phase [25], shifts the regulatory balance away from σS-dependent stress tolerance and toward motility and metabolic flexibility.

Together, these findings identify *yobF-cspC* as a major adaptive hotspot associated with increased motility and enhanced acetate utilization in aging colonies.

#### Mutations affecting glutamate metabolism and acid resistance

Genome analysis revealed a second mutational axis involving genes of the glutamate metabolic network and the glutamate-dependent acid resistance system (AR2), a major survival strategy in acidic environments [47].

The most striking example is strain Y12, which carried a large IS*5*-mediated deletion spanning 14 contiguous genes and eliminating the entire AR2 module, including the master regulators *gadE*,* gadF*,* gadW*,* gadX*, the periplasmic chaperones *hdeA*,* hdeB*,* hdeD*, and the *mdtEF* efflux operon [48, 49]. Additional mutations affected regulators or transporters of the glutamate–γ-aminobutyric acid (GABA) axis: Y17 carried an S184R substitution in the DNA-binding domain of *rcsB* [50]; Y4 harbored a *gabP* missense mutation (Gly272Val) [51], affecting a proton-driven GABA transporter involved in glutamate/GABA flux; and Y15, Y22, and Y23 contained intergenic mutations located between the glutamate transporter genes (*gltT* or *gltP*) [52] and neighboring loci.

Functionally, AR2 protects cells by converting glutamate to GABA via GadA/B and exporting it through GadC in exchange for extracellular glutamate, thereby consuming protons and stabilizing cytoplasmic pH [53–55]. Key components of this system are typically induced during stationary phase through σ^S^ [56] while the regulator RcsB, mutated in Y17, cooperates with GadE to activate AR2 genes and represses flagellar regulators such as flhD [53–55].

These mutations collectively suggest selective downregulation of energetically costly acid resistance. Consistent with this interpretation, Y12, despite lacking AR2 entirely, retained high competitiveness, implying that suppression of acid resistance conserves energy and resources in the structured environment of an aging colony.

Furthermore, the strong competitive success of Y4, which combines a *cspC* mutation with a *gabP* substitution, highlights the potential for synergistic interactions between stress-response disruption and altered glutamate flux. By contrast, isolates carrying mutations near *gltT* or *gltP* (Y22, Y23) showed no measurable fitness advantage.

Together, these findings indicate that adaptive trade-offs in aging colonies involve coordinated remodeling of glutamate metabolism and stress tolerance, driven by mutations in key regulators and transport systems. Both AR2 disruption and *cspC* loss reduce σ^S^-dependent stress responses, but through distinct mechanisms, an important point given the partly RpoS-independent effects of *cspC* mutations.

#### Mutations in *ydcI*: a distinct route for metabolic and stress-response rewiring

Genomic analysis also revealed mutations in stress-related regulators, but their adaptive outcomes differed markedly. Notably, strain Y3 carries a single nonsense mutation in *ydcI*, a LysR-type transcription factor involved in pH homeostasis and acetate metabolism [57]. This single mutation confers a strong fitness advantage, as evidenced by its high CIC in mixed-colony competition assays (Fig. 2; Table 1; [23]), making Y3 the second most competitive isolate after Y4, and importantly, an isolate whose adaptive success arises independently of *cspC*.

Mechanistically, YdcI represses *gltA* (citrate synthase) and *ackA* (acetate kinase), thereby limiting TCA cycle flux and acetate turnover [57–59]. Its inactivation likely enhances acetate assimilation and energy production. Beyond metabolism, recent work [60] demonstrates that YdcI also contributes directly to intracellular pH regulation: it promotes cytoplasmic acidification under alkaline stress and modulates σ^S^-dependent pathways through activation of *iraP* (σ^S^ regulator*)*. Consistent with this dual metabolic and pH rewiring, Y3 exhibits robust growth on acetate, reduced biofilm formation, and increased swarming motility (Figs. 1 and 2; [23]).

Strain Y16, which carries mutations in *ygfK*, *ydbK*, and *cspC*, also displays elevated competitiveness. Among these, *ygfK* [61] encodes a predicted oxidoreductase strongly upregulated during colony aging (Supplementary Table S1), suggesting a contribution to oxidative stress adaptation. However, it is difficult to estimate its specific adaptive contribution as its phenotype likely reflects the combined effects of multiple mutations.

By contrast, other stress-related mutations, such as those in *htpX* (Y1) or *uspC* (Y25), did not confer measurable fitness benefits, underscoring the specificity of adaptive targets.

Together, these findings identify *ydcI* as a key locus driving competitive success through combined metabolic and pH rewiring, revealing an adaptive route that operates independently of *cspC* yet converges functionally on reduced σ^S^-dependent stress responses.

#### Cell-envelope mutations: specialization and antibiotic resistance

In addition to stress- and metabolism-related variants, we identified mutations affecting cell envelope and morphology genes. Strain Y8, carrying a conservative E290D substitution in *fliI*, showed enhanced swarming motility and high competitive fitness (Figs. 1 and 2; [23]), consistent with increased efficiency in flagellar export and surface motility [62–64].

Strain Y2, which did not outperform the wild type in colony competition assays, carried a unique C→T substitution in *mrdB* (*rodA*), a peptidoglycan glycosyltransferase essential for lateral wall synthesis, commonly mutated in experimental evolution [65, 66]. Importantly, the resulting A234T mutation has been previously linked to β-lactam resistance, particularly ampicillin [67]. This phenotype was confirmed in our earlier work, which showed a markedly increased MIC for ampicillin in Y2 using automated Vitek 2 antibiograms [23]. Thus, colony aging can generate clinically relevant gain-of-function mutations even in the absence of direct selection or measurable fitness advantage. Other resistance-associated mutations were also detected in *rpoB* (T563P in Y22 and Y25; see above), conferring rifamycin resistance without affecting colony competitiveness.

Overall, these findings show that colony aging produces not only adaptive variants enhancing motility, metabolism, and competitiveness, but also specialized mutants such as antibiotic-resistant strains. This highlights that mutations without an immediate fitness benefit in colonies may still carry ecological, evolutionary, or clinical significance, reinforcing the idea that aging colonies can act as reservoirs of functionally relevant diversity [23].

### Transcriptomic rewiring in *yobF-cspC* mutants

#### Global expression changes Y5/Y6 vs. WT

Mutations in the *yobF-cspC* operon were the most frequent genomic alterations among evolved strains and were associated with enhanced swarming, acetate assimilation, and competitive fitness, prompting us to examine their transcriptomic consequences. We compared the transcriptomes of isolates Y5 and Y6, which exhibited a high colony competitive index (CIC) (Table 1; Fig. 2), with the wild-type strain under identical conditions (7-day-old colonies, two biological replicates, each with five technical replicates). Both strains carried insertion sequence elements within the *yobF* coding sequence (IS*1* in Y5 and IS*5* in Y6; Supplementary Figure S2A), close to the internal cryptic promoter *cspCp8* [38, 68]. Sanger resequencing confirmed the structure and boundaries of these insertions (Supplementary Figure S2A-B).

The operon is transcribed from three σ⁷⁰-dependent promoters: *yobFp1*, *yobFp2*, and the intragenic cryptic promoter *cspCp8* [38, 68], with σ⁷⁰ encoded by the housekeeping sigma factor *rpoD* [69]. Transcriptomic analysis showed marked downregulation of both *yobF* and *cspC* in Y5 and Y6 compared with WT (Supplementary Figure S2C), consistent with repression of the operon. Because the insertions disrupt the *yobF* coding sequence in both isolates, production of a full-length YobF protein is unlikely despite a low but detectable transcript signal.

To identify expression changes common to both isolates, we analyzed Y5 and Y6 jointly against WT. This approach captures regulatory effects driven by *cspC* silencing rather than isolate-specific mutations. Differential expression analysis (LIMMA [70]) identified 88 significantly upregulated and 61 significantly downregulated genes (|log₂FC| ≥ 0.58, padj ≤ 0.05; Supplementary Figure S3). To investigate broader regulatory patterns, we applied Weighted Gene Co-expression Network Analysis (WGCNA) [71] to the most variable genes (424 genes, including the 149 DEGs), which identified two major co-expression modules (Supplementary Figures S5-S6).

##### Module 1: upregulated metabolic and biosynthetic pathways

**Fig. 4:**
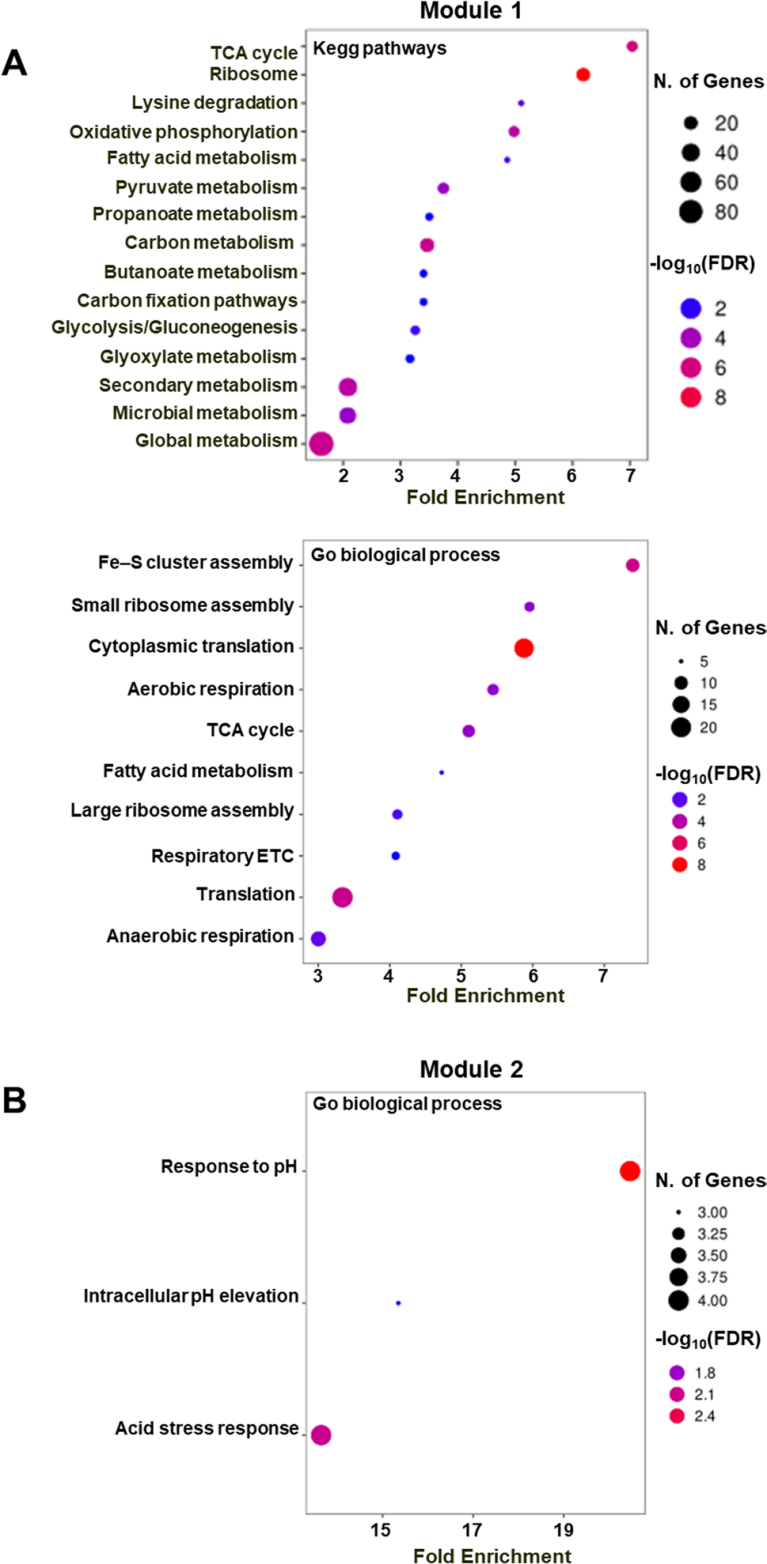
Functional enrichment of WGCNA modules. **A** Module 1 (243 genes), negatively correlated with the wild-type (WT), showing coordinated activation of metabolic and biosynthetic functions. KEGG pathway enrichment includes the TCA cycle, glyoxylate metabolism, oxidative phosphorylation, fatty-acid metabolism, and ribosomal proteins. GO biological process enrichment highlights cytoplasmic translation, Fe–S cluster assembly, and aerobic respiration. **B** Module 2 (133 genes), positively correlated with WT, is enriched in GO biological processes related to acid stress response and pH homeostasis. For both panels, bubble size represents the number of genes per category, and color intensity corresponds to –log₁₀(FDR)

Module 1 (243 genes; Supplementary Tables S7-S8) showed a strong negative correlation with WT (eigengene *r* = -0.75, *p* = 1.6 × 10⁻⁷⁷) and contained all 88 upregulated DEGs (Supplementary Tables S3-S4, Supplementary Figure S4). Hub-gene analysis identified 44 highly connected, significantly upregulated genes (padj 2.5 × 10⁻⁵ to 0.04). Functional enrichment revealed broad activation of central metabolism (Fig. 4A, Supplementary Figures S7-S8), including the TCA and glyoxylate cycles, pyruvate and fatty-acid metabolism, oxidative phosphorylation, and ribosomal protein biosynthesis. Upregulated genes (e.g., *aceE*,* lpd*,* sucA*,* acnA*,* sdhA*,* fumC*) indicate enhanced oxidative metabolism and carbon recycling.

Enrichment of arginine/proline and polyamine catabolic pathways (FDR < 0.01), including *puuA*,* puuD*,* astA*, and *speB*, suggests remodeling of nitrogen and polyamine fluxes supporting pH buffering via GABA production [72, 73]. Increased expression of Fe–S cluster assembly and anaerobic respiration genes indicates redox flexibility suited to oxygen gradients within colonies. Upregulation of *uxuA*,* uxuB*, and *uxaC* supports hexuronate utilization and glyoxylate replenishment.

Ribosomal protein enrichment (FDR = 5.24 × 10⁻¹⁰) and downregulation of *hpf* (log₂FC = − 0.39, padj = 2.0 × 10⁻³) indicate that Y5/Y6 maintain active translation rather than forming hibernating 100S ribosomes [74, 75], consistent with their elevated CIC. Together, these changes define a transcriptional state optimized for sustained metabolism and active growth under nutrient-limited conditions.

##### Module 2: downregulated acid and stress responses

Module 2 (133 genes; Supplementary Tables S9–S10) was positively correlated with WT (*r* = 0.84, *p* = 5.0 × 10⁻¹¹⁴) and contained most downregulated DEGs (Supplementary Tables S5-S6, Supplementary Figure S4). Functional enrichment analyses (Fig. 4B and Supplementary Figure S9) showed strong repression of the glutamate-dependent acid-resistance system (AR2) [47] and pH homeostasis genes. Overrepresented categories included “response to pH” and the *GadX/GadW* regulons (FDR < 10⁻⁸), spanning *gadA/BC*,* gadC*,* gadE*,* hdeA/B*, and *slp*. Additional downregulated genes included *cbpA* (co-chaperone), *aidB* (detoxification), *ahpC* (oxidative-stress response), and bssS (biofilm regulation), consistent with the low biofilm index and high motility of Y5/Y6. Repression of the *tna* operon (*tnaA*,* tnaC*), which produces indole and ROS, may further reduce oxidative stress [23, 76].

Together, the LIMMA and WGCNA analyses reveal a coordinated transcriptional shift in Y5/Y6: activation of central metabolic and biosynthetic programs coupled with repression of acid-resistance, detoxification, and oxidative-stress responses.

#### Linking CspC-regulated targets to transcriptome reprogramming

To explore whether the transcriptional changes observed in Y5/Y6 strains reflect known CspC targets, we compared our WGCNA modules with published CLIP-seq datasets for CspC [77]. In extraintestinal pathogenic *E. coli* (ExPEC), this analysis identified approximately 681 mRNA targets spanning diverse functional categories. The same study also reported ~ 713 putative CspC targets in *E. coli* K-12 MG1655, although without detailed functional annotation.

Among the 243 genes of Module 1, 86 overlapped with CspC targets (expected ≈ 41.5; 2.1× enrichment; hypergeometric test, *p* = 8.8 × 10⁻¹³), indicating a strong enrichment of putative direct targets, particularly genes involved in translation and respiration. Module 2 showed a weaker but still significant enrichment (31/133 genes; expected ≈ 22.8; hypergeometric test, *p* = 0.04). Notably, none of the acid-resistance genes of the RpoS-dependent AR2 system (e.g., *gadA/B/C*,* gadE*) were among the overlapping targets (Supplementary Tables S7 and S9).

These enrichments suggest that the adaptive advantage conferred by *cspC* disruption arises from disruption of specific regulatory subnetworks, largely independent of RpoS, particularly those involving in ribosome biogenesis and central metabolism. This reorganization appears to favor biosynthetic and respiratory programs that enhance survival in aging colonies.

To validate these insights experimentally, we next assessed whether *a cspC* deletion could reproduce the adaptive phenotype observed in evolved isolates, and whether its effects depended on σˢ.

### Fitness analyses of Δ*cspC *and rpoS* mutants

Transcriptomic analyses indicated that *yobF-cspC* mutations are associated with broad reprogramming of stress and metabolic responses, accompanied by enhanced swarming motility and fitness. To test whether loss of *cspC* alone is sufficient to reproduce this adaptive phenotype, we constructed isogenic Δ*cspC* mutants with or without fluorescent reporters, derived from the WT strain, and competed them against a WT strain expressing mKate under aging colony conditions. To determine whether the effect of Δ*cspC* was mediated solely through its action on σ^S^, we also generated double mutants rpoS* Δ*cspC* from strain NEC291, which carries a *rpoS*359::Tn10 insertion (see Materials and Methods, Table 2).

**Fig. 5:**
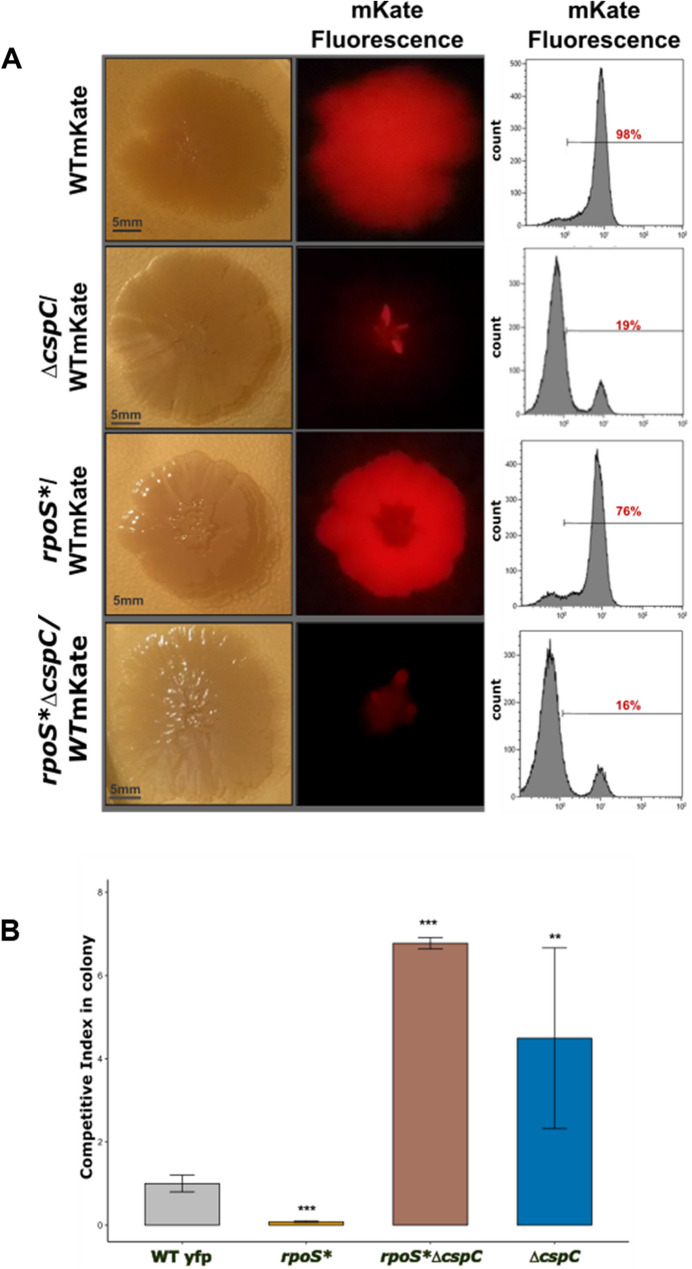
Epistasis between *cspC* and *rpoS* in colony competitiveness. **A** Representative 1:1 mixed colonies of *E. coli* WT-mKate with the indicated mutants (Δ*cspC*, rpoS*, rpoS* Δ*cspC*) imaged under visible light (left; scale bars = 5 mm) and mKate fluorescence (middle). Corresponding flow-cytometry histograms (right) show the proportion of mKate-positive cells within each mixed colony. **B** Colony competitive index (CIC) of mutant strains relative to the WT reference strain (mean ± SD, *n* = 4). CIC values were normalized to control mixtures of WT-YFP / WT-mKate, set to 1. Statistical significance was assessed on log₂-transformed CIC values using two-tailed one-sample t-tests against zero (corresponding to neutral competitiveness). Parallel Wilcoxon signed‑rank tests gave similar conclusions. Significance levels are indicated as follows: *** *p* < 0.001, ** *p* < 0.01; * *p* < 0.05

Competitions were initiated by co-inoculating 1:1 mixtures of mutants and WT on LB agar filters (Fig. 5A). Colony composition was quantified after 7 days by fluorescence imaging and flow cytometry. CIC values were calculated as described in [23]. To assess epistatic interactions between *cspC* and *rpoS*, CIC were analyzed on log₂-transformed values, providing a symmetric measure of relative fitness (Fig. 5B). These values were tested against zero.

rpoS* mutants displayed a marked fitness defect (mean log₂[CIC] = − 3.66 ± 0.16; one-sample t-test, t₃ = −27.64, *p* = 1.0 × 10⁻⁴). In contrast, Δ*cspC* mutants exhibited a strong competitive advantage (mean log₂[CIC] = 2.52 ± 0.58; t₃ = 9.35, *p* = 2.6 × 10⁻³). Remarkably, the rpoS* Δ*cspC* double mutant showed a similarly high fitness (mean log₂[CIC] = 2.76 ± 0.03; t₃ = 230, *p* = 1.8 × 10⁻⁷), indicating that *cspC* deletion fully compensates for the loss of σˢ activity. Wilcoxon signed-rank tests yielded consistent qualitative conclusions.

**Fig. 6:**
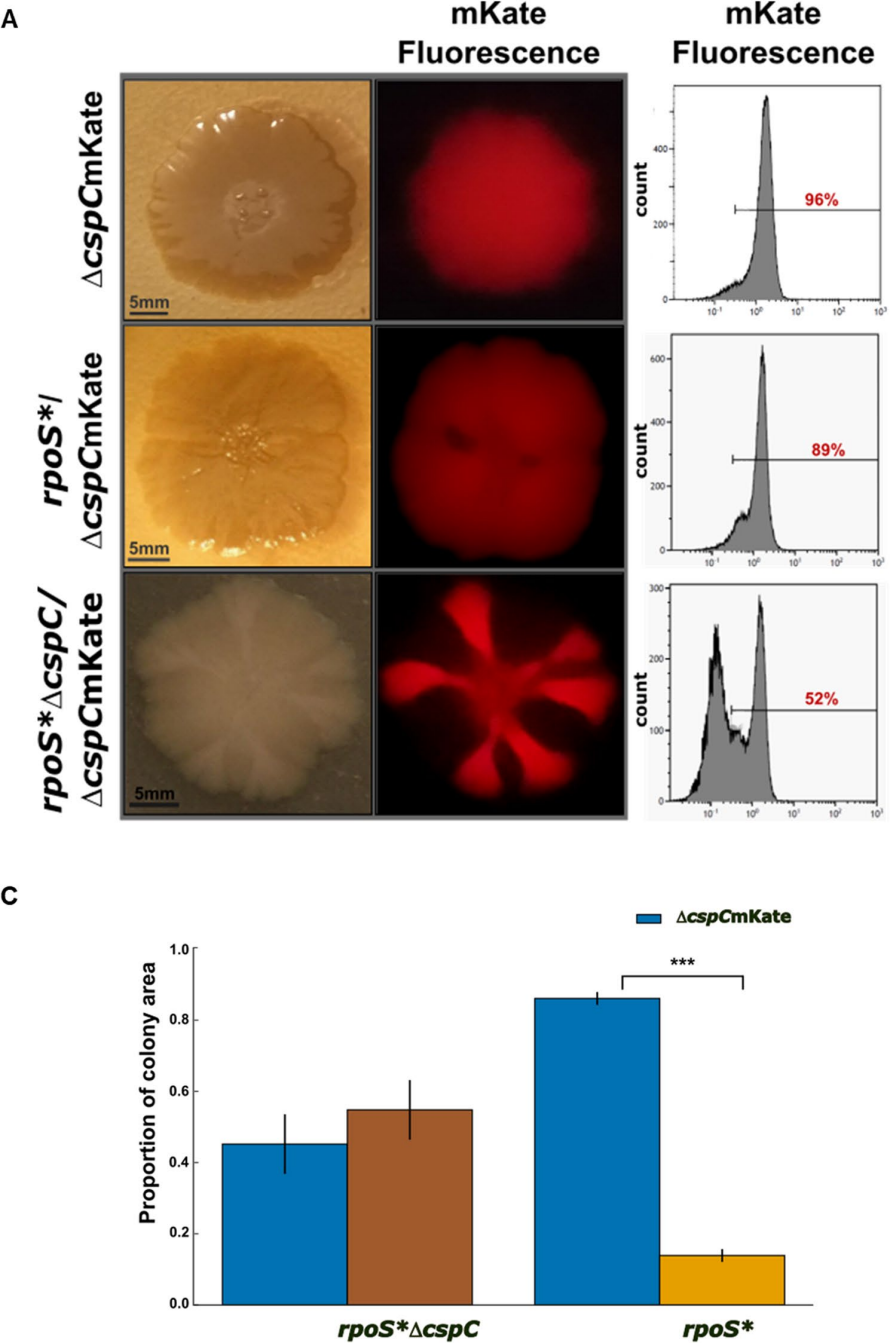
Competition between Δ*cspC* mKate, rpoS*, and rpoS* Δ*cspC* mutants in mixed colonies. **A** Representative 1:1 mixed colonies of ΔcspC mKate with either rpoS* or rpoS* ΔcspC mutants. Colonies were imaged under visible light (left; scale bars = 5 mm) and mKate fluorescence (middle). Flow-cytometry histograms (right) show the proportion of ΔcspCmKate–positive cells in each mixed colony. **B** Quantification of competition outcomes based on the proportion of colony area occupied by each strain (mean ± SD; *n* = 4 colonies per condition). Δ*cspC*mKate (blue) dominated rpoS* (orange), occupying 86.1% ± 1.8% of the colony area. In contrast, Δ*cspC*mKate and rpoS* Δ*cspC* (brown) occupied comparable proportions. The mean ± SD values are shown on the original proportion scale for interpretability, while statistics were computed on log₂‑transformed ratios (log₂[Δ*cspC*/rpoS*] and log₂[Δ*cspC*/rpoS*Δ*cspC*]) using two-tailed one-sample t-tests against zero. Wilcoxon signed-rank tests yielded consistent qualitative conclusions. *** *p* < 0.001

To further probe this interaction, we performed direct pairwise competitions between Δ*cspC*-mKate and rpoS* strains, and between Δ*cspC*-mKate and rpoS* Δ*cspC* strains (Fig. 6A).

To account for the compositional nature of strain proportions (summing to 1), competition outcomes were analyzed using log₂-transformed fitness ratios (Δ*cspC*/competitor), which provide an unconstrained measure of relative fitness. These values were tested against zero.

Δ*cspC* strongly dominated rpoS* strains (mean log₂ ratio = 2.64 ± 0.22; one-sample t-test, t₃ = 24.25, *p* = 1.5 × 10⁻⁴), corresponding to an approximately sixfold fitness advantage. In contrast, the log₂ ratio for Δ*cspC* versus rpoS* Δ*cspC* did not differ from zero (mean = − 0.28 ± 0.49; t₃ = −1.15, *p* = 0.33), indicating equivalent competitive fitness (Fig. 6B). Loss of *cspC* thus restores fitness even in an *rpoS*-deficient background, highlighting σˢ-independent mechanisms through which this RNA chaperone contributes to adaptation in aging colonies.

## Discussion

In this study, we investigated how cells diversify into phenotypically distinct lineages within aging *E. coli* colonies. The characterization of 24 isolates from a 3-week-old colony revealed strain-specific profiles but recurrent traits, including enhanced swarming motility, reduced biofilm formation, and improved acetate utilization, consistent with a shift toward motile, high-metabolism phenotypes in nutrient-limited colonies. Whole-genome sequencing uncovered striking mutational heterogeneity among isolates, yet with clear convergence on genes involved in transcriptional regulation, stress responses, and envelope homeostasis, predominantly through IS‑mediated events [65].

Among these targets, the *yobF-cspC* operon was mutated in half of the isolates, suggesting that its disruption represents a major adaptive route under nutrient-limited, spatially structured growth conditions. Recent work by Shimada et al. demonstrated that in *E. coli*, this operon is expressed from σ⁷⁰-dependent promoters under nitrogen-responsive control via NtrC [78]. Under nitrogen-replete conditions, NtrC represses these promoters, whereas nitrogen limitation activates NtrC (NtrC ~ P), leading to induction of *cspC* transcription. This regulatory configuration places *cspC* at the interface between nitrogen sensing and global stress regulation.

Building on this regulatory framework, aging bacterial colonies are expected to experience progressive nutrient depletion, including nitrogen limitation as biomass accumulates and cellular turnover increases. Sustained NtrC activation under such conditions would therefore promote *cspC* expression, potentially reinforcing stress-associated transcriptional programs through stabilization of targets such as *rpoS* mRNA [36, 77]. Recurrent inactivation of *cspC* in independently evolved isolates may thus represent adaptive uncoupling from nitrogen starvation signaling. By attenuating nitrogen-driven reinforcement of stress maintenance pathways, these mutations could shift the balance toward metabolic reactivation, enhanced respiration, and improved competitive fitness in resource-depleted, spatially structured environments.

This interpretation is consistent with the regulatory architecture described by Shimada et al. [78], although nitrogen flux and NtrC activity were not directly measured in this study. It thus provides a plausible ecological framework for the repeated selection of *cspC* mutations.

In agreement with this model, transcriptomic analyses of isolates Y5 and Y6 show that *cspC* disruption is accompanied by marked induction of central metabolic and biosynthetic pathways, coupled to repression of stress networks, particularly acid resistance, defining a transcriptional state biased toward metabolic plasticity at the expense of protective functions. Integrating these data with fitness analyses and functional reconstructions highlights complementary roles of CspC and RpoS in shaping adaptation: deletion of *rpoS* substantially reduces competitiveness, underscoring its importance for survival in aging colonies; partial attenuation of the RpoS regulon, as in isolate Y12 carrying deletions in the AR2 acid‑resistance system, can nevertheless be beneficial; and, strikingly, additional deletion of *cspC* in an *rpoS*‑deficient strain restores high fitness, consistent with σ^S^-independent gains, potentially driven by metabolic activation.

Together, these observations support a dual adaptive model in aging colonies, in which partial suppression of stress responses, particularly acid resistance, improves resource allocation, whereas *cspC* inactivation provides a metabolic advantage that can bypass σˢ‑dependent regulation. In this framework, *cspC* disruption may not merely attenuate stress signaling but rather reconfigures the transcriptome toward enhanced acetate utilization, respiratory activity, anabolic functions, and motility.

Notably, previous studies have also observed fitness gains associated with *cspC* disruption in laboratory-evolved *E. coli* strains [37], supporting the evolutionary relevance of this regulatory shift.

Additional lines of evidence reinforce this interpretation. First, CspC mRNA targets [77] are strongly enriched among WGCNA Module 1 genes, which include ribosomal and TCA cycle functions. Second, AR2 acid-resistance genes in Module 2 (canonical RpoS targets) are not direct CspC binders.

Further transcriptomic profiling of additional *yobF-cspC* mutants, including clean deletions, would strengthen the generalizability of this regulatory signature beyond isolates Y5 and Y6.

Preliminary data from isolate Y4 also support this. Y4 combines a 66-bp *cspC* deletion with a *gabP* missense mutation [51]. It displays high CIC, enhanced motility, and improved acetate utilization (Fig. 3). A clean *gabP* deletion alone did not increase fitness but appears to amplify the benefits of *cspC* disruption in mixed-colony assays.

Other adaptive routes in the 24 isolates illustrate both convergence and diversity. Beyond metabolic and regulatory rewiring, we detected gain-of-function mutations associated with drug resistance (*mrdB* A234T, *rpoB* T563P). These highlight colony aging as a generator of variants with ecological and clinical significance, even without direct selective pressure.

Mutations/deletions affected acid resistance (*gad*), nitrogen flux regulation (*gabP*,* gltP*,* gltS*), and stress regulators (*rcsB*,* rpoA*,* rpoB*,* ydcI*,* cspE*,* mrdB*,* fliI*), reflecting varied evolutionary paths. Some *cspC* mutants, like Y19, showed reduced competitiveness, suggesting an influence of epistatic interactions or genomic context. Conversely, isolates without detectable mutations, such as Y20, may persist by occupying specific ecological niches or relying on metabolic exchanges with evolved lineages. This indicates that cooperative dynamics and spatial segregation also contribute to stabilizing diversity within aging colonies.

These findings echo long-term stationary-phase (LTSP) experiments [79], where *E. coli* adapts under chronic starvation through recurrent mutational trajectories, although the fitness advantage of each mutation often depends on the genetic background in which it arises. They are also consistent with recent spatial analyses showing that colonies are physiologically heterogeneous, with distinct subpopulations distributed across microenvironments [12]. The interaction of spatial heterogeneity, nutrient recycling, and historical contingency likely underlies the recurrent yet context-dependent outcomes observed here.

## Conclusion

Our previous work revealed that aging *E. coli* colonies contain spatially restricted survivor islands composed of metabolically evolved specialists [23]. These active subpopulations could, in more complex environments such as biofilms or microbiomes, contribute to substantial microbial heterogeneity and potentially create diagnostic blind spots in clinical settings [80, 81].

Here, whole-genome sequencing of 24 evolved isolates revealed extensive mutational diversity, suggesting that aging colonies act as powerful incubators of evolutionary diversification. Alongside a clear convergence on the *yobF-cspC* locus, we also uncovered distinct adaptive routes involving acid-resistance regulators, glutamate transporters, and motility genes. Crucially, several isolates acquired clinically relevant gain-of-function phenotypes, such as antibiotic resistance emerging in the absence of selective pressure.

Overall, our results link genetic evolution, regulatory rewiring, and fitness gains in structured bacterial populations. Downregulation *of cspC* emerged as a recurrent route to a metabolic phenotype, and could operate independently of σ^S^. This shift redirected adaptation from stress resistance toward metabolic flexibility, which proved strongly advantageous under nutrient limitation.

In clinical contexts, similar regulatory rewiring may help explain how resilient subpopulations arise within biofilms and chronic infections, and could contribute to long-term persistence. Targeting metabolic plasticity and diversification processes may thus offer opportunities to weaken these persistent bacterial subpopulations.

This study was performed in a single laboratory model (aging colonies of *E. coli* K-12 MG1655 on LB agar) and is based on 24 evolved isolates, which may limit the generality and representativeness of the observed evolutionary trajectories. In addition, while replay competition assays provide controlled measurements of relative fitness, they do not fully reproduce the spatial structure, metabolic gradients, and prolonged selective pressures operating during three weeks of colony aging. Future work should extend sampling and test whether similar metabolic and regulatory rewiring occurs in other taxa and microbial communities, as well as in mature biofilms or in vivo infection models.

## Materials and methods

### Bacterial strains and constructions

All strains used in this study are derivatives of *Escherichia coli* MG1655 [82]. Detailed strain descriptions are provided in Table 2.

**Table 2:**
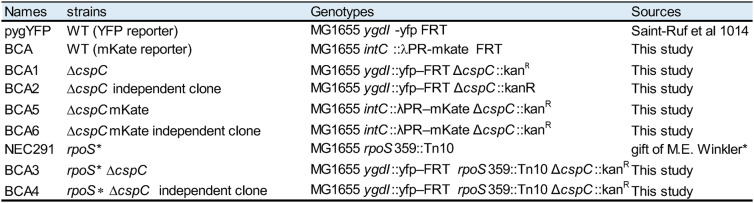
Bacterial strains used in this study. All strains are derivatives of *Escherichia* *coli* MG1655. Fluorescent reporters were integrated at *ygdI::yfp-FRT* (YFP) or *intC*::λPR–mKate–FRT (mKate). The allele *rpoS* corresponds to *rpoS*359::Tn10. Independent clones correspond to separate recombination events obtained from the same construction

The 24 evolved isolates analyzed here were recovered from a 3-week-old colony derived from the parental strain pygYFP, which carries the *ygdI*::yfp-FRT reporter cassette and served as the wild-type (WT) reference. pygYFP and the evolved isolates were described previously in [23].

A second WT reporter strain expressing mKate was generated by integrating the red fluorescent reporter λPR–mKate at the intC locus (*intC*::λPR–mKate).

The rpoS* strain used in this study carries the *rpoS*359::Tn10 allele [23].

The single mutant strains Δ*cspC*, as well as the double mutants rpoS* Δ*cspC*, were constructed by P1vir transduction [83] of the Δ*cspC* allele from the Keio collection [84] into either the YFP WT background or the mKate WT background. When required, the kanamycin resistance cassette (kanᴿ) was excised using Flp recombinase expressed from plasmid pCP20 [85]. All genetic modifications were verified by PCR.

### Media and growth conditions

*E. coli* strains were routinely grown in Luria–Bertani (LB) broth or on LB agar (Difco, Lawrence, KS, USA) at 37 °C with aeration. When required, antibiotics were added at the following final concentrations: ampicillin (100 µg ml⁻¹), chloramphenicol (30 µg ml⁻¹), and kanamycin (50 µg ml⁻¹).

For colony growth experiments, approximately 200 cells from overnight LB cultures were spotted onto polycarbonate membrane filters (pore size 0.22 μm; diameter 13 or 25 mm; GE Water and Process Technologies, Trevose, PA, USA) placed on fresh LB agar plates (15 g l⁻¹ agar, 20 ml per 85-mm Petri dish). Plates were incubated at 37 °C under controlled humidity to limit evaporation, using aluminum foil wrapping. Colonies grown on membrane filters were easily transferred with sterile forceps and, when required for RNA extraction or flow cytometry, filters were immersed in 1 ml of LB or PBS and vortexed to resuspend all cells.

### DNA isolation, genome sequencing, and mutation analysis

Genomic DNA was prepared from overnight cultures by centrifugation (4,472 × g, 10 min, 4 °C) and washing in PBS. DNA was extracted using lysozyme/SDS lysis followed by phenol–chloroform extraction [86], proteinase K digestion, ethanol precipitation, and final purification on spin-columns. DNA quality and concentration were assessed using a NanoDrop spectrophotometer and an Agilent 2100 Bioanalyzer, according to GATC Biotech requirements.

Genomic DNA was sequenced by GATC Biotech (Konstanz, Germany) using an Illumina HiSeq 2000 platform. Depending on the batch, resequencing was performed using paired-end 2 × 46 bp reads, or de novo sequencing was performed using standard and mate-pair libraries with 2 × 50 bp reads. Raw sequencing data were quality controlled using FastQC (v0.11.9) [87] and fastp (v0.20.0) [88]. Reads with Phred quality score ≥ Q20 were retained after trimming, with QC summaries generated using MultiQC (v1.7) [89].

In all cases, raw FASTQ files were delivered and subsequently reanalyzed using breseq v0.37.1 [27] for read alignment, variant calling, and annotation. Mutation detection was performed in consensus mode using default parameters, with *Escherichia coli* K-12 MG1655 (RefSeq NC_000913.3 [82]) as reference. For isolates sequenced in multiple libraries or lanes, paired-end FASTQ files were provided simultaneously to breseq, which internally combined reads for mutation calling. All mutations identified by whole-genome sequencing in each evolved isolate were independently validated by Sanger resequencing.

### Estimation of the Ka/Ks ratio (non-synonymous/synonymous mutations)

To estimate the Ka/Ks ratio, we applied three approaches, two of which accounted for mutation biases specific to aging colonies. Let *i* denote the mutation class (AT→CG, AT→GC, AT→TA, CG→GC, CG→TA, GC→TA), *si* the observed number of synonymous mutations, *ni* the non-synonymous mutations (including stop codons), *Si* the total possible synonymous sites, and *Ni* the non-synonymous sites. Ka/Ks was computed as:

1. (Σn_i_/ΣN_i_) / (Σs_i_/ΣS_i_) = 1.63 (no mutational bias correction),
2. Σn_i_ / Σ(N_i_·s_i_/S_i_) = 2.29 (mutational bias correction using synonymous sites),
3. Σ(S_i_·n_i_/N_i_) / Σs_i_ = 2.54 (mutational bias correction using non-synonymous sites).

The first estimate is crude, whereas the latter two account for mutational biases, though the second is limited by the low number of synonymous substitutions.

### Sanger validation of sequence variants in the *yobF-cspC* locus

To confirm the insertions or deletions identified by breseq, the *yobF-cspC* locus and its flanking regions (positions 1,906,441–1,908,276 in *E. coli* K-12 MG1655; RefSeq NC_000913.3) were PCR-amplified using primers P*yob*F*cspC*1 (5′-CCTTCCAGTTGTTCTGCATGAGGT-3′) and P*yob*F*cspC*2 (5′-GGTCGCCGAACGATAATCCTT-3′), yielding a 1.8-kb product in the wild-type strain. PCRs were performed using standard Taq DNA polymerase (Thermo Fisher Scientific) in a Bio-Rad T100 thermocycler. Amplification products were gel-purified and sequenced by GATC Biotech (Konstanz, Germany) using an ABI Prism 3730xl DNA Analyzer with BigDye Terminator v3.1 chemistry (Applied Biosystems, Foster City, CA, USA). Sequencing primers included P*yob*F*cspC*1, P*yob*F*cspC*2, and two internal primers located near *cspC* (P*cspC*1: 5′-ATCAGTGGATCCTATCAGATAGCTGTTACG-3′; P*cspC*2: 5′-GAATTTTTCATATGGCAAAGATTAAAGGTC-3′).

### RNA extraction and transcriptome profiling

RNA-seq experiments were performed in biological duplicates from 7-day-old colonies of isolates Y5, Y6, and the parental strain *E. coli* pygYFP (hereafter referred to as WT) [23].

Total RNA was extracted using the RNeasy Mini Kit (Qiagen) with on-column DNAse treatment, following the manufacturer’s instructions. RNA integrity and purity were verified using a NanoDrop spectrophotometer and an Agilent 2100 Bioanalyzer. Complementary DNA (cDNA) was synthesized with SuperScript™ II Reverse Transcriptase (Thermo Fisher Scientific) and random hexamers.

Hybridization was carried out by Roche NimbleGen (Madison, WI, USA) on *E. coli* K-12 MG1655 whole-genome ORF arrays (RefSeq GCF_000005845.2), as previously described [90, 91]. Labeling, hybridization, scanning, and RMA normalization (background correction and quantile normalization) were performed by NimbleGen according to standard protocols. Normalized, non-log₂ intensity data were used for all subsequent analyses.

### Bioinformatic analysis

Differential expression was assessed with the LIMMA package (Bioconductor v3.21 [92]) in R v4.0.5 [93] within RStudio [94]. Genes with an adjusted *p*-value (padj) ≤ 0.05 (Benjamini–Hochberg correction) and |log₂FC| ≥ 0.58 were considered significantly differentially expressed. Weighted Gene Co-expression Network Analysis (WGCNA v1.73) [71] was applied to identify groups of co-regulated genes and hub genes across WT, Y5, and Y6 transcriptomes. Modules were optimized for functional coherence based on eigengene correlations and network connectivity.

### Functional enrichment and network analysis

Functional enrichment of differentially expressed genes was assessed using ShinyGO v0.76 [31] with default parameters and a false discovery rate (FDR) threshold of 0.05, based on the *E. coli* K-12 MG1655 background.

Protein-protein interaction networks were generated with STRING v11.5 [32] using a minimum confidence score of 0.4. STRING networks were subsequently imported into Cytoscape v3.9.1 [95] for visualization. Enriched GO terms and network visualizations are shown in Fig. 4 and Supplementary Figures S3, S6-S8.

### Assessment of phenotypic traits

Phenotypic data for swarming motility, biofilm formation index (BFI), and growth on acetate were obtained from our previous study [23], which provides detailed experimental protocols and measurements.

Briefly, swarming motility was quantified on 0.5% LB agar plates after 18 h at 37 °C as the colony surface area. Biofilm formation was assessed using the BioFilm Ring Test (BioFilm Control, Saint-Beauzire, France) following the manufacturer’s recommendations, and growth on acetate was evaluated using Biolog GN2 microplates (Biolog, Hayward, CA, USA) as the ratio of OD₆₀₀ after 24 h at 37 °C relative to the parental strain.

### Competition assays and CIC calculations

The relative fitness of mutant strains (Δ*cspC*, rpoS* Δ*cspC)* was determined in mixed-colony competition assays against the parental pygmkate strain.

Overnight cultures were adjusted to 10⁵ live cells ml⁻¹, with viability confirmed by flow cytometry after propidium iodide staining. Mutant and parental cells were mixed at a 1:1 ratio and deposited on LB agar filters as described above. As a control, a 1:1 mixture of the parental pygYFP and pygmkate strains was used. After 4 or 7 days of incubation at 37 °C, colonies were imaged under YFP and mKate fluorescence filters. The relative proportions of strains were quantified by measuring fluorescent colony areas using ImageJ/Fiji.

The colony competitive index (CIC) was calculated as:

$$CIC=\frac{{A}_{mutant}/{A}_{pygmkate}}{{CIC}_{pa}}$$

where A denotes the fluorescent area, and CIC_pa is the mean ratio A_pygYFP/A_WT mkate obtained from control colonies [23].

Additional pairwise competition assays compared Δ*cspC* with rpoS* Δ*cspC* and rpoS* strains. For these assays, Δ*cspC*-mKate was co-inoculated with its competitor at a 1:1 ratio on LB agar. After 4 days of incubation, colonies were imaged under visible and mKate fluorescence, and strain areas were quantified with ImageJ. Statistical analysis of competition outcomes are described in the Statistical analyses section.

### Flow cytometry experiments

Cell suspensions were analyzed using a Gallios 10-color, 4-laser flow cytometer (Beckman Coulter, Brea, USA).

YFP fluorescence was detected in the FL1 channel (488 nm excitation, 525/20 nm bandpass filter), and mKate fluorescence in the FL3 channel (488 nm excitation, 620 nm long-pass filter).

Samples were acquired at approximately 2,000 events s⁻¹, and 50,000 events were recorded per sample. Data were collected using Kaluza software (Beckman Coulter) and analyzed with either Kaluza or FlowJo (FlowJo LLC, Ashland, OR, USA).

### Statistical analyses

All statistical analyses were performed using R (v4.0.5) [93] within RStudio [94]. Statistical methods not described in the bioinformatic analysis sections are detailed below.

Correlations between phenotypic traits and competitive fitness (Fig. 1) were assessed using Spearman’s rank correlation coefficients (two-sided tests), as the data did not necessarily meet normality assumptions and monotonic rather than strictly linear relationships were expected. Correlation analyses were performed on 24 evolved strains. Exact *p*-values are reported.

Comparisons between isolates carrying mutations in the *yobF–cspC* locus and isolates without such mutations (Fig. 3) were performed using two-sided Welch’s t-tests on log₂-transformed values. Welch’s test was selected to account for potential inequality of variances between groups.

For competition assays (Figs. 5 and 6), relative fitness was quantified either as colony competitive index (CIC) or as the proportion of colony area occupied by each strain in mixed colonies. Because strain proportions are compositional and therefore not statistically independent, competition outcomes were analyzed using log₂-transformed fitness ratios (log₂[CIC] or log₂[Δ*cspC*/competitor]), providing an unconstrained and symmetric measure of relative fitness.

Log₂-transformed values were tested against zero using two-sided one-sample t-tests. For each comparison, the test statistic (t), degrees of freedom (df), and exact *p*-values are reported.

Given the small sample size (*n* = 4 independent colonies per condition), Wilcoxon signed-rank tests were additionally performed as a non-parametric robustness check. These analyses yielded consistent qualitative conclusions.

## Supplementary Information

Additional file 1: Supplementary Figures. This file contains supplementary Figures S1–S9 providing additional analyses supporting the main text, including transcriptomic comparisons, network analyses, and functional enrichment results.

Additional file 2: Supplementary Tables S1-S2. Supplementary Table S1. List of genes mutated in Escherichia coli strains isolated from 3-week-old colonies. The table summarizes the mutation types and the strains in which they were identified. The column “RpoS regulon” indicates whether each gene belongs to the RpoS regulon or encodes a protein involved in the regulation of RpoS activity. Transcriptomic data from [23] are included, showing log2 fold changes between 7-day and 1-day colonies (D7 vs D1) and the corresponding adjusted *p*-values (Storey’s correction). Supplementary Table S2. GO biological process enrichment analysis of mutated genes from 24 Escherichia coli strains.

Additional file 3: Supplementary Tables S3-S6. Supplementary Table S3. Differentially expressed genes upregulated in Y5/Y6 compared with WT, identified by LIMMA analysis. The table reports gene identifiers, genomic positions, functional annotations, log₂ fold changes, adjusted *p*-values (FDR), and LIMMA statistics. Supplementary Table S4. Pathway enrichment analysis of genes upregulated in Y5/Y6 compared with WT. Enrichment analyses were performed using KEGG, RegulonDB, and Gene Ontology (biological process) databases. The table reports adjusted *p*-values (FDR), fold enrichment, number of genes per pathway, and the corresponding gene lists. Supplementary Table S5. Differentially expressed genes downregulated in Y5/Y6 compared with WT, identified by LIMMA analysis. Supplementary Table S6. Pathway enrichment analysis of genes downregulated in Y5/Y6 compared with WT (RegulonDB and GO biological processes).

Additional file 4: Supplementary Tables S7-S10. Supplementary Table S7. Genes belonging to WGCNA Module 1 (turquoise), negatively correlated with the WT condition. The table lists gene identifiers, functional annotations, differential expression results (log2 fold change, Y5/Y6 vs WT, and LIMMA adjusted p-values), and WGCNA network metrics, including kME (module membership; correlation between gene expression and the module eigengene), GS (gene significance; correlation with the trait of interest), and kWithin (intramodular connectivity). Hub genes are indicated. The table also reports whether each gene was identified as a CspC target based on CLIP-seq data [77]. Supplementary Table S8. Pathway enrichment analysis of genes belonging to the WGCNA turquoise module. Enrichment analyses were performed on genes assigned to the turquoise module using KEGG, EcoCyc, RegulonDB, and Gene Ontology (biological process) databases. The table reports significantly enriched pathways, the number of module genes associated with each pathway, pathway gene counts, fold enrichment values, and false discovery rate (FDR), adjusted p-values. Complete lists of contributing genes are provided for each pathway. Supplementary Table S9: Genes of WGCNA Module 2 (blue module), positively correlated with WT. The table lists gene identifiers, functional annotations, differential expression statistics (log2 fold change, Y5/Y6 vs WT, and LIMMA adjusted p-values), and WGCNA network metrics (kME, GS, kWithin; as defined in Supplementary Table S7), and whether each gene is reported as a CspC target based on CLIP-seq data [77]. Supplementary Table S10. Pathway enrichment analysis of genes belonging to the WGCNA module 2. Enrichment analyses were performed on genes assigned to the module 2 (blue) using RegulonDB and Gene Ontology (biological process) databases. The table reports significantly enriched regulatory pathways and biological processes, the number of module genes associated with each pathway, pathway gene counts, fold enrichment values, and false discovery rate (FDR)-adjusted p-values. Complete gene lists contributing to each enrichment are provided.

Additional file 5: Unprocessed PCR gel images for Supplementary Figure S2B.

## Data Availability

Raw and processed transcriptomic data have been deposited in the Gene Expression Omnibus (GEO) under accession numbers GSE312419 and GSE312426. Whole-genome sequencing reads are available in the NCBI Sequence Read Archive (SRA) under BioProject PRJNA1355247. Sanger sequencing data confirming the IS insertion in strains Y5 and Y6 have been deposited in GenBank (accession numbers PX635285 and PX635286). All other resequencing traces, additional datasets, and detailed analysis scripts, including software versions and parameter settings, are available from the corresponding author upon reasonable request.

## Abbreviations

AR2: glutamate-dependent acid resistance system
BFI: biofilm formation index
CIC: colony competitive index
CLIP-seq: cross-linking immunoprecipitation sequencing
DEG(s): differentially expressed gene(s)
FDR: false discovery rate
GABA: gamma-aminobutyric acid
GEO: Gene Expression Omnibus
GO: Gene Ontology
Ka/Ks: ratio of non-synonymous to synonymous substitutions
KEGG: Kyoto Encyclopedia of Genes and Genomes
LB: Luria–Bertani
LIMMA: Linear Models for Microarray Data
LTSP: long-term stationary phase
ORF: open reading frame
PBS: phosphate-buffered saline
PCA: principal component analysis
PPI: protein–protein interaction
RMA: robust multi-array average
ROS: reactive oxygen species
SNS: single-nucleotide substitution
SRA: Sequence Read Archive
STRING: Search Tool for the Retrieval of Interacting Genes/Proteins
TCA cycle: tricarboxylic acid cycle
WGCNA: weighted gene co-expression network analysis
WT: wild type
YFP: yellow fluorescent protein
σS (σˢ): stationary-phase sigma factor (RpoS)
