# Supplementary material for: Genomic diversification, adaptive convergence, and regulatory rewiring in aging Escherichia coli colonies
Source: BMC Microbiol. 2026 Mar 31;26:455. doi: 10.1186/s12866-026-04983-z (PMC13162491; doi:10.1186/s12866-026-04983-z)
Supplement: Supplementary file 1 — Additional file 1: Supplementary Figures. This file contains supplementary Figures S1–S9 providing additional analyses supporting the main text, including transcriptomic comparisons, network analyses, and functional enrichment results. [file 12866_2026_4983_MOESM1_ESM.pdf]

Title: Genomic diversification, adaptive convergence, and regulatory rewiring in aging Escherichia coli colonies

Authors : Claude Saint-Ruf, Adrien Launay, Olivier Tenaillon, Ivan Matic

Additional file 1

Supplementary Figures

Supplementary Figure S1: Network and functional enrichment analysis of mutated genes

Supplementary Figure S2: Structural, PCR, and expression characterization of the *yobF-cspC* locus in Y5 and Y6 isolates

Supplementary Figure S3: PCA and differential expression analysis of WT versus Y5/Y6 isolates

Supplementary Figure S4: Functional enrichment of differentially expressed genes (DEGs)

Supplementary Figure S5: Weighted gene co-expression network analysis (WGCNA) of transcriptomes

Supplementary Figure S6: Top 15 differentially expressed genes in WGCNA modules (limma; Y5/Y6 vs WT) (barplots)

Supplementary Figure S7: Protein-protein interaction (PPI) network of Module 1 genes (turquoise)

Supplementary Figure S8: Protein-protein interaction (PPI) network of Module 1 hub genes

Supplementary Figure S9: Protein-protein interaction (PPI) network of Module 2 genes (blue)

Supplementary Figure S1. Network and functional enrichment analysis of mutated genes

(A)STRING protein-protein interaction (PPI) network showing predicted functional associations among all genes mutated across the 24 evolved isolates. The network (STRING v12.0; confidence ≥ 0.4) was visualized in Cytoscape (v3.10.0). Nodes represent proteins encoded by mutated genes, and edges indicate experimentally validated or predicted interactions. Functional clusters correspond mainly to transcriptional regulators, stress-response genes, and envelope-associated proteins.

(B) Functional enrichment analysis (GO biological processes) of mutated genes performed with ShinyGO. The dot plot shows the top enriched GO terms. Bubble size represents the number of genes per category, and color indicates statistical significance (-$\log_{10}$ FDR).

(C)Distribution of mutation types identified by whole-genome sequencing of evolved *E. coli* strains**.** Illumina sequencing data were analyzed using Breseq (Deatherage and Barrick, 2014). The plot shows the number of single-nucleotide substitutions (SNS), insertion events (IS element insertions), short sequence insertions, and deletions. Each substitution type is represented separately. Insertion types are grouped regardless of IS family or orientation.

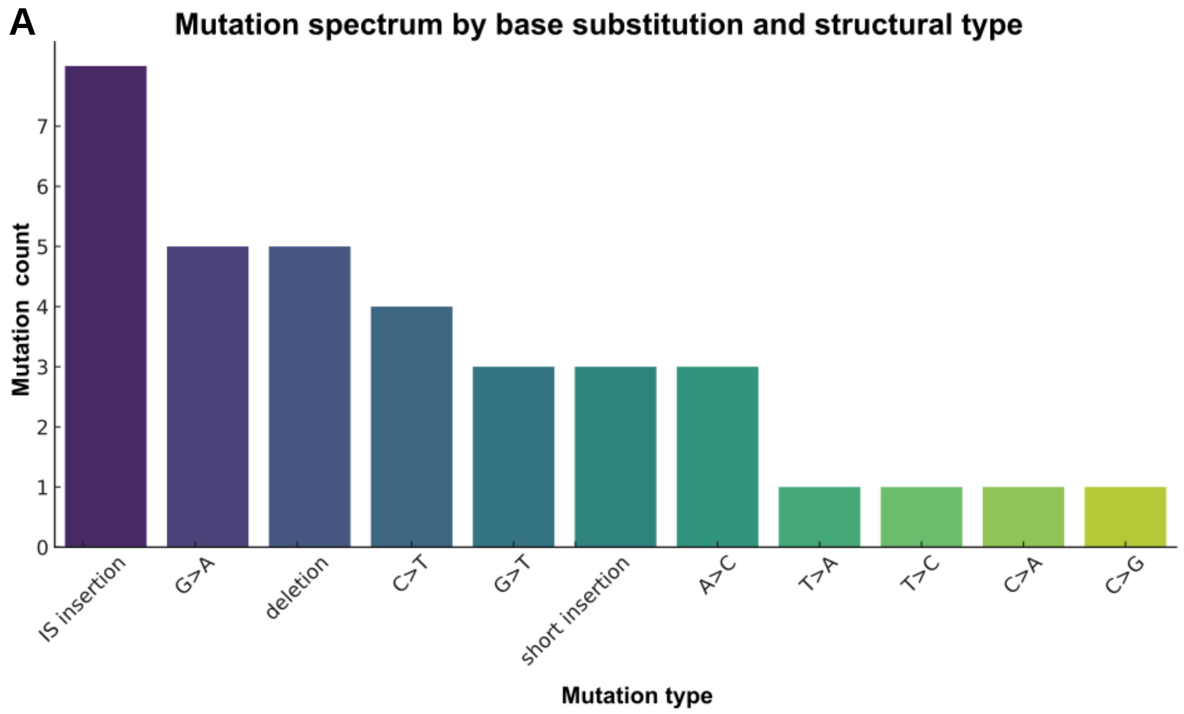

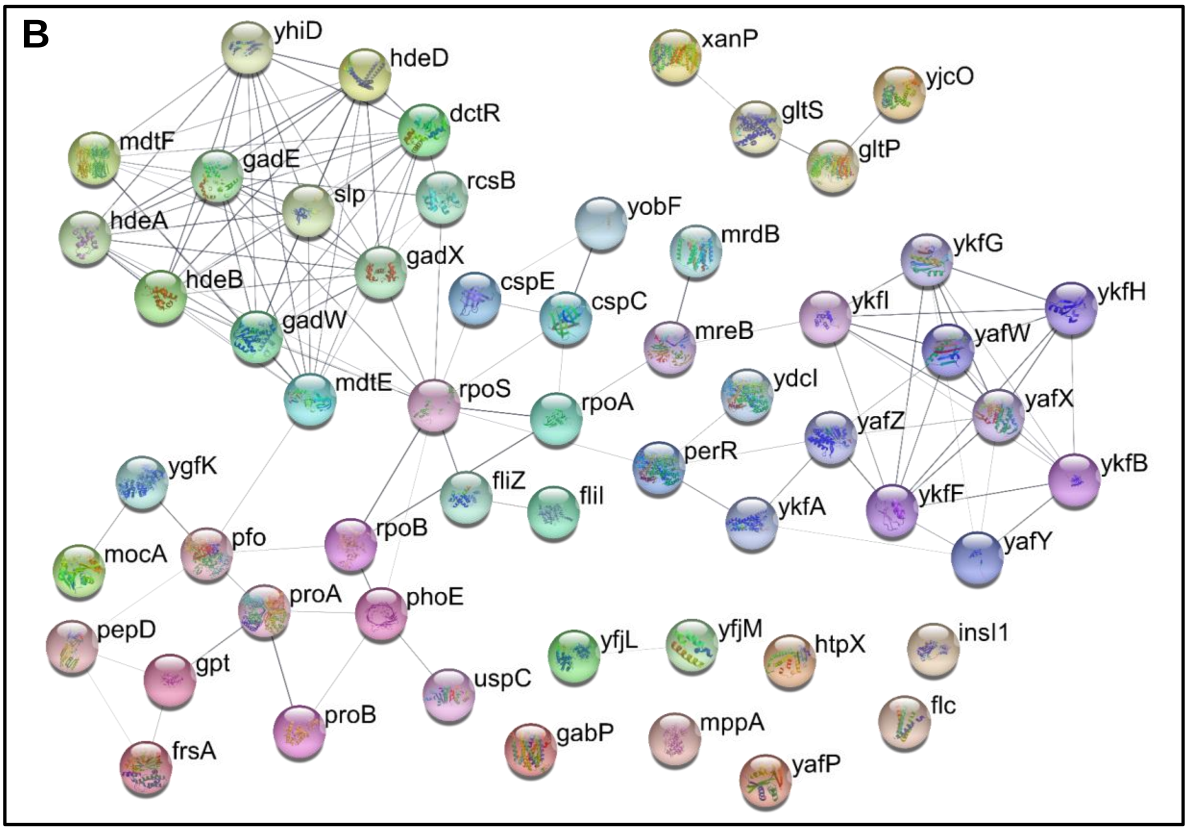
B
yhiD
hdeD
dctR
mdtF
gadE
rcsB
slp
hdeA
gadX
hdeB
gadW
mdtE
rpoS
xanP
yjcO
gltS
gltP
yobF
mrdB
cspE
cspC
mreB
rpoA
ykfG
ykfH
ykfI
yafW
ydcI
yafX
yafZ
perR
ykfB
ygfK
fliZ
fliI
ykfA
ykfF
yafY
mocA
pfo
rpoB
proA
phoE
pepD
gpt
frsA
proB
uspC
yfjL
yfjM
htpX
insI1
flc
gabP
mppA
yafP

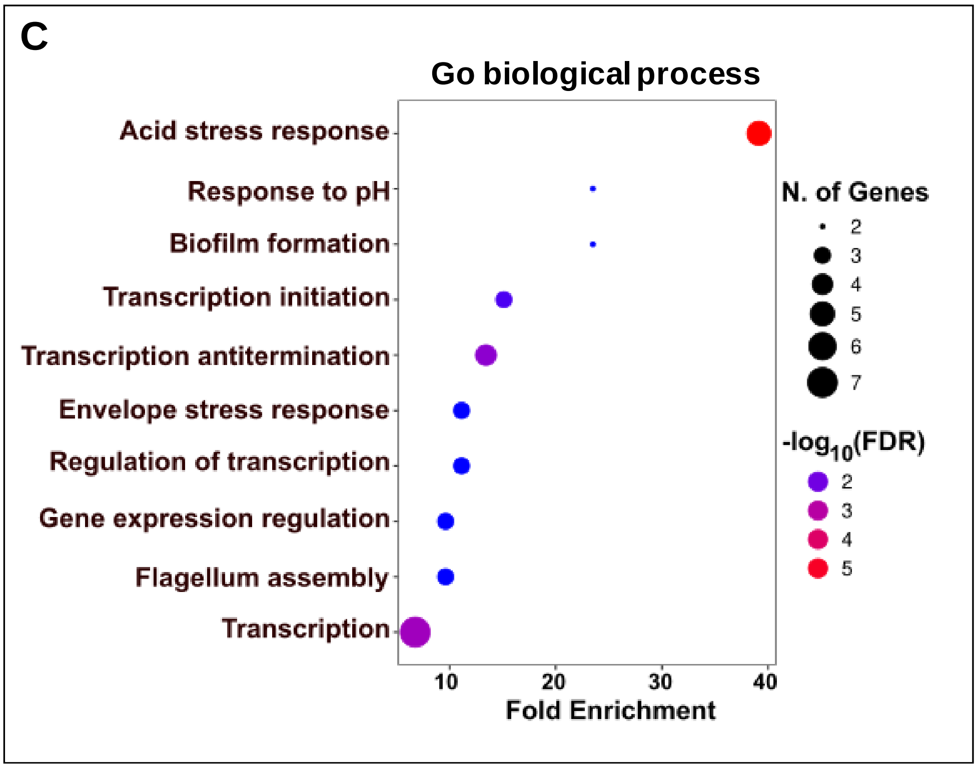
C
Go biological process
Acid stress response
Response to pH
Biofilm formation
Transcription initiation
Transcription antitermination
Envelope stress response
Regulation of transcription
Gene expression regulation
Flagellum assembly
Transcription
10
20
30
40
Fold Enrichment
N. of Genes
2
3
4
5
6
7
-log10(FDR)
2
3
4
5

Supplementary Figure S2. Structural, PCR, and expression characterization of the *yobF-cspC* locus in Y5 and Y6 isolates

(A) Schematic representation of the *yobF-cspC* locus in isolates Y5 and Y6 showing IS insertions. In Y5, an IS1 element (698 bp, insAB transposase + 70 bp of flanking regions) is inserted at position 1,907,482 with a 9 bp target site duplication, in the opposite orientation to the operon. In Y6, an IS5 element (980 bp, insH transposase + 280 bp of flanking regions) is inserted at position 1,907,444 with a 4 bp duplication in the same region. Sanger sequences confirming the IS1 and IS5 insertions have been deposited in GenBank under accession numbers PX635285 and PX635286.

(B) PCR validation of the *yobF-cspC* locus in WT, Y5, and Y6 isolates using primers PyobFcspC1 and PyobFcspC2. The expected WT fragment (1.8 kb) and the larger amplicons in Y5 (≈2.6 kb) and Y6 (≈3.1 kb) confirm the IS1 and IS5 insertions, respectively. M: DNA size marker (1 kb ladder). PCR products were gel-purified and confirmed by Sanger sequencing (see Methods). Uncropped and unprocessed gel images are provided in Additional file 5.

(C) Expression of the *yobF-cspC* operon in WT, Y5 and Y6 *E. coli* colonies. Normalized microarray expression values for *grpE*, *cspC*, and *yobF* are shown for the wild-type (WT), Y5, and Y6 isolates. Differential expression in this panel was assessed using LIMMA on log2-transformed expression values, with biological replicate as a blocking factor (duplicateCorrelation; 2 biological replicates per strain, each with 5 technical replicates). P-values were adjusted using the Benjamini–Hochberg procedure across the six targeted tests shown in this panel (three genes × two pairwise comparisons: Y5 vs WT and Y6 vs WT). Both Y5 and Y6 show downregulation of *cspC* and *yobF* compared with WT (adjusted p-values: Y5 vs WT: *cspC* 0.00595, *yobF* 1.1×10^{-9}; Y6 vs WT: *cspC* 2.5×10^{-5}, *yobF* 8.9×10^{-4}), whereas *grpE* remains unchanged. Bars represent median normalized expression values with interquartile range (IQR) from 10 replicate arrays per strain. Expression values were obtained using Roche NimbleGen microarrays and are shown in arbitrary units (a.u.).

Supplementary Figure S2

A

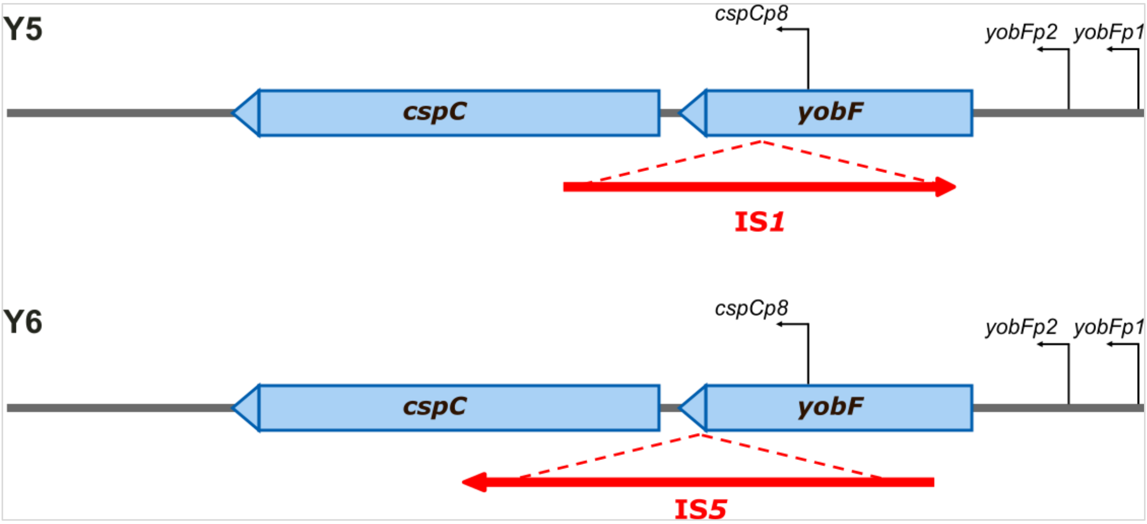

B

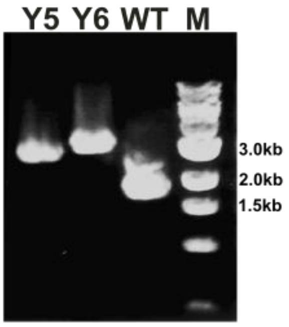

C

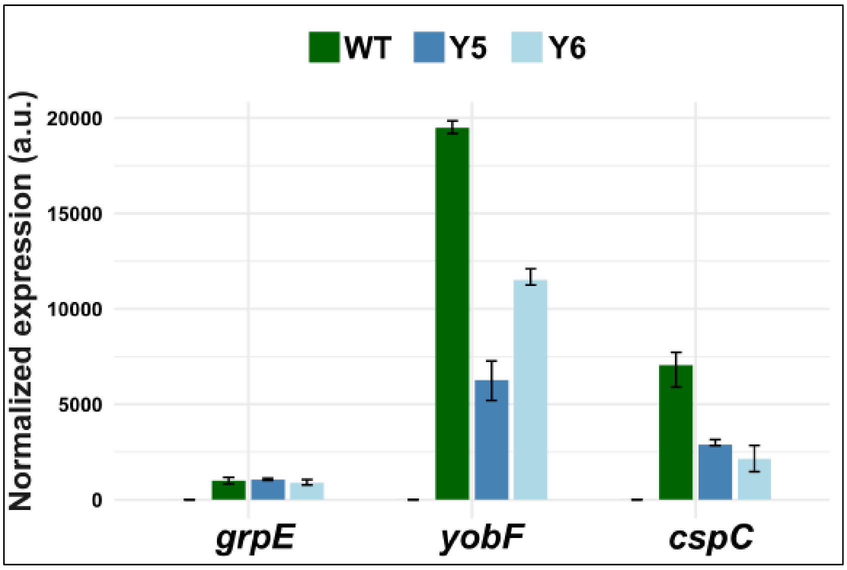

Supplementary Figure S3. PCA and differential expression analysis of WT versus Y5/Y6 isolates

(A) Principal component analysis (PCA) of WT, Y5 and Y6 transcriptomes.

PCA was performed on log_2-transformed and scaled microarray expression values from 7-day-old colonies. Each point represents the average transcriptomic profile obtained from five technical replicates for each biological sample.

PC1 accounted for 52.1% of the total variance and clearly separated WT samples (green) from the evolved Y5 (dark blue) and Y6 (light blue) isolates.

PC2 explained an additional 29.4% of the variance and captured within-group variability.

Each point represents one biological replicate.

A

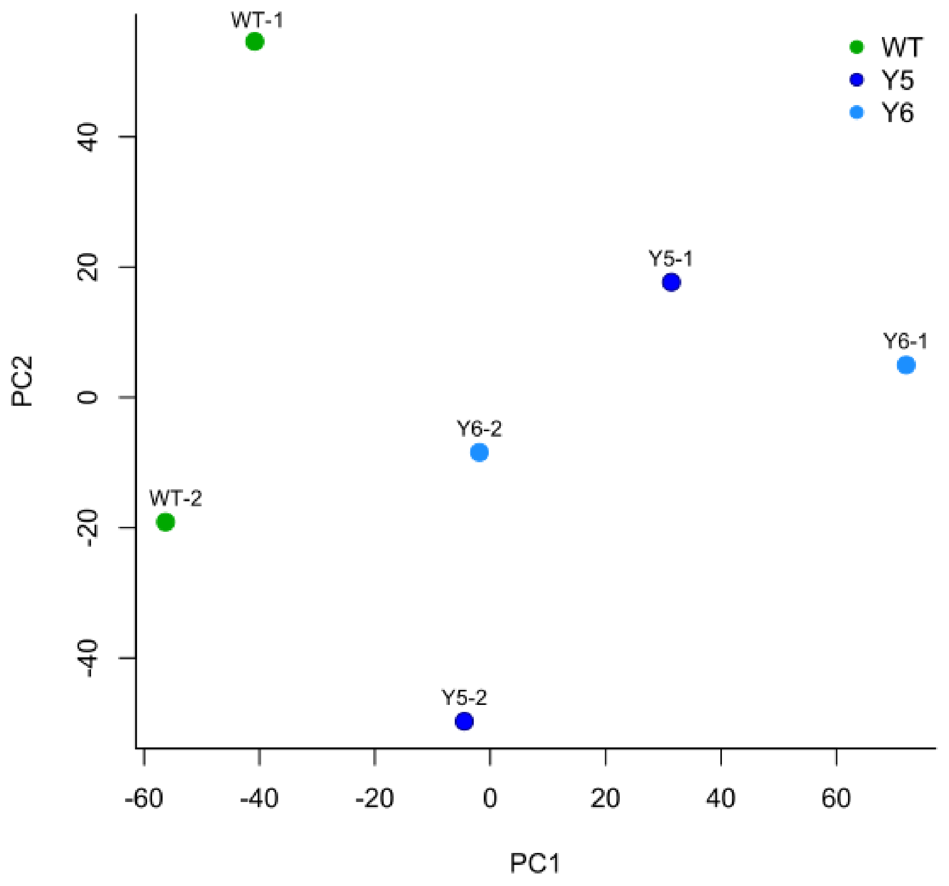

(B) Volcano plot of differential gene expression (LIMMA) comparing Y5/Y6 isolates versus WT.

The plot displays all genes detected in the transcriptomic analysis, with $\log_2$ fold change on the x-axis and $-\log_{10}$ adjusted p-value on the y-axis. Differential expression analysis was performed using a 1.5-fold change threshold ($|\log_2 FC| > 0.585$) and padj < 0.05. In the figure, genes highlighted in red correspond to the most significantly deregulated transcripts, meeting the stricter criteria padj < 0.05 and $|\log_2 FC| > 1$. The names of the top DEGs are annotated.

B

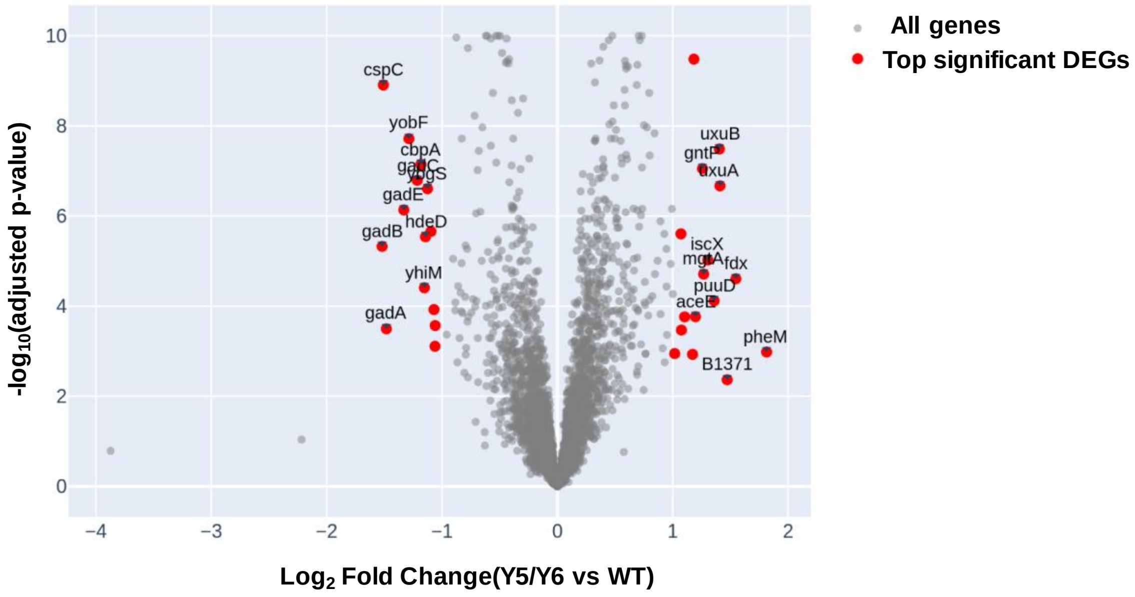

Supplementary Figure S4. Functional enrichment of differentially expressed genes (DEGs)

Dot plots of KEGG and GO biological process enrichment for genes

(A) up-regulated (89 genes) and (B) down-regulated (62 genes) in Y5/Y6 compared to WT colonies, as identified by LIMMA. Each dot represents a significantly enriched pathway, with size proportional to the number of genes and color reflecting statistical significance ($-\log_{10}(FDR)$). Complete pathway lists are provided in Supplementary Tables S4-S6.

A

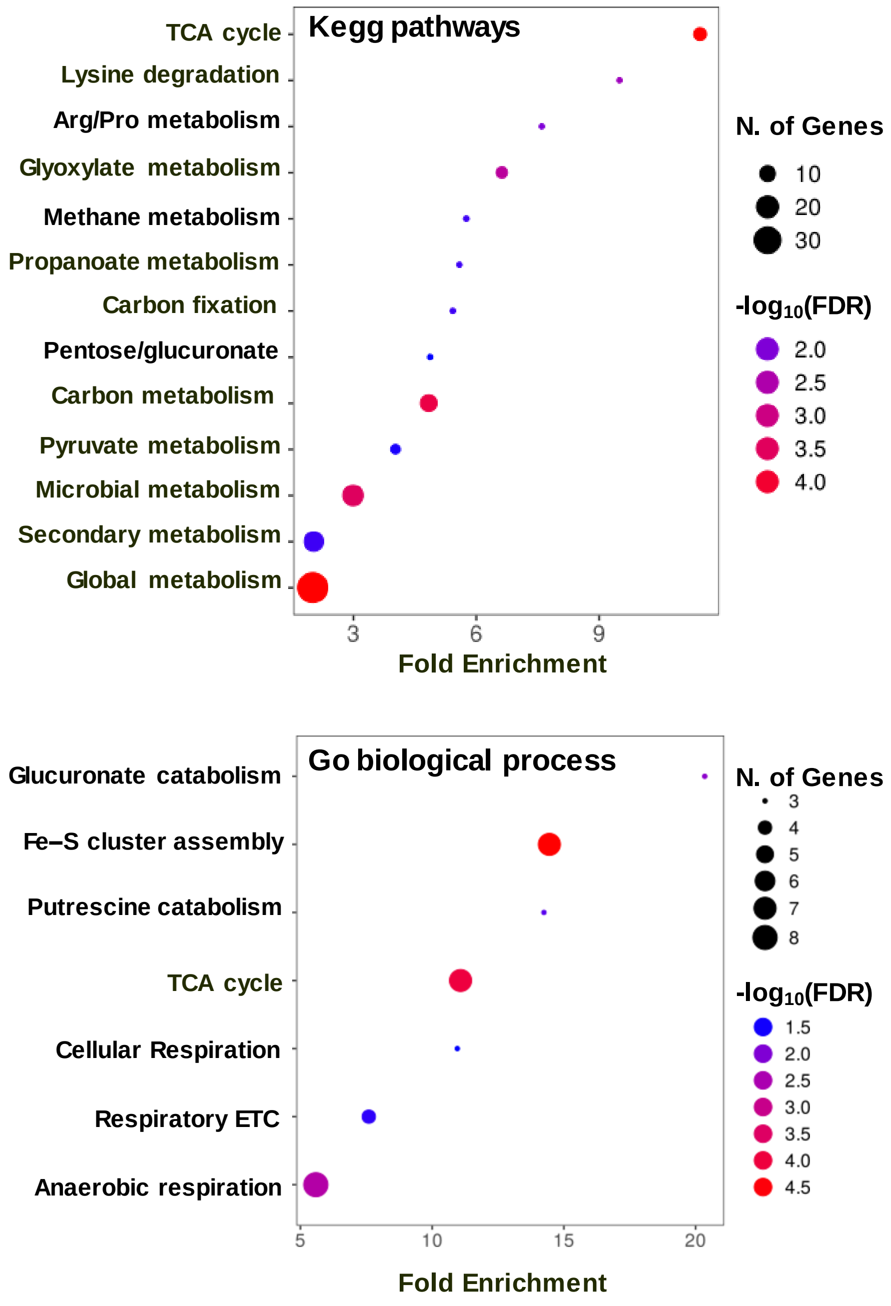

B DEGs down in Y5/Y6 versus WT

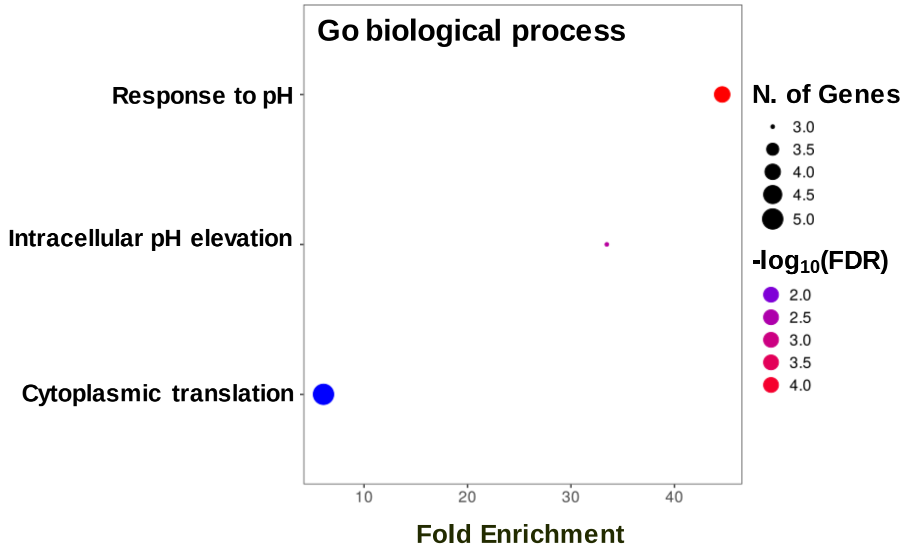

Supplementary Figure S5. Weighted gene co-expression network analysis (WGCNA) of transcriptomes

(A) Hierarchical clustering dendrogram of genes across WT and Y5/Y6 transcriptomes. Three co-expression modules were detected: the turquoise module (257 genes), the blue module (122 genes), and the brown module (44 genes). The grey module shown in the figure contains only one gene. The brown module was not further analyzed due to lower significance. The turquoise and blue modules correspond to Module 1 and Module 2, respectively. After gene reassignment based on kME, kWithin, and GS values, Module 1 contained 243 genes and Module 2 contained 134 genes.

(B) Module–trait relationship heatmap showing eigengene (ME) correlations with experimental conditions (Y5/Y6 versus WT). Each cell displays the correlation coefficient and the associated *p*-value.

(C) Eigengene boxplots: Boxplots depicting log2 fold change distributions in each module indicating overall module expression changes. Outlier gene symbols are labeled. A Wilcoxon rank-sum test was used to assess statistical significance between the two distributions (p-value shown).

Supplementary Figure S5

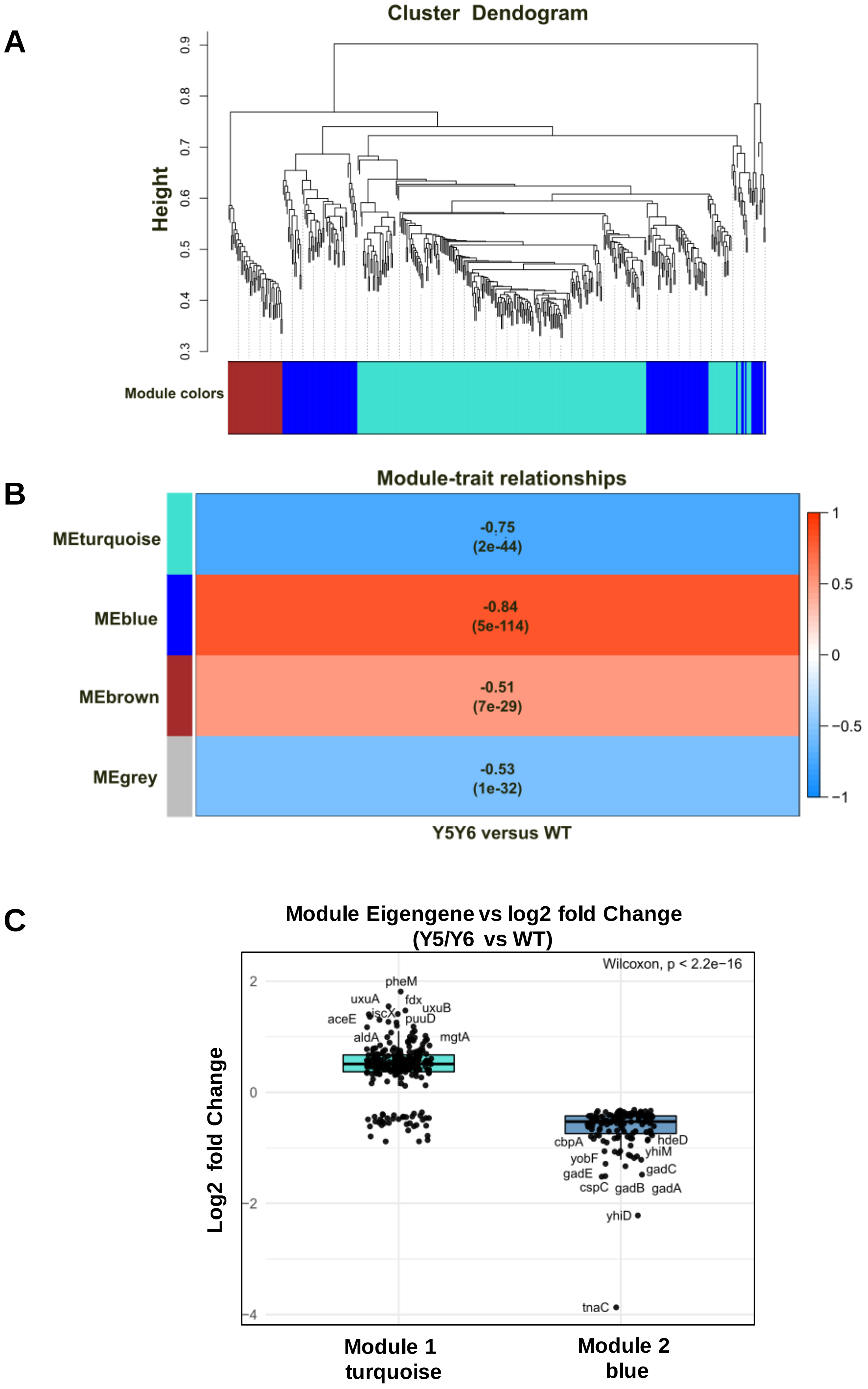

Supplementary Figure S6. Top 15 differentially expressed genes in WGCNA modules (limma; Y5/Y6 vs WT) (barplots)

Barplots show the top 15 genes ranked by LIMMA log2 fold change (Y5/Y6 vs WT) for two WGCNA modules: Module 1 (negatively correlated with WT; genes upregulated in Y5/Y6) and Module 2 (positively correlated with WT; genes downregulated in Y5/Y6).

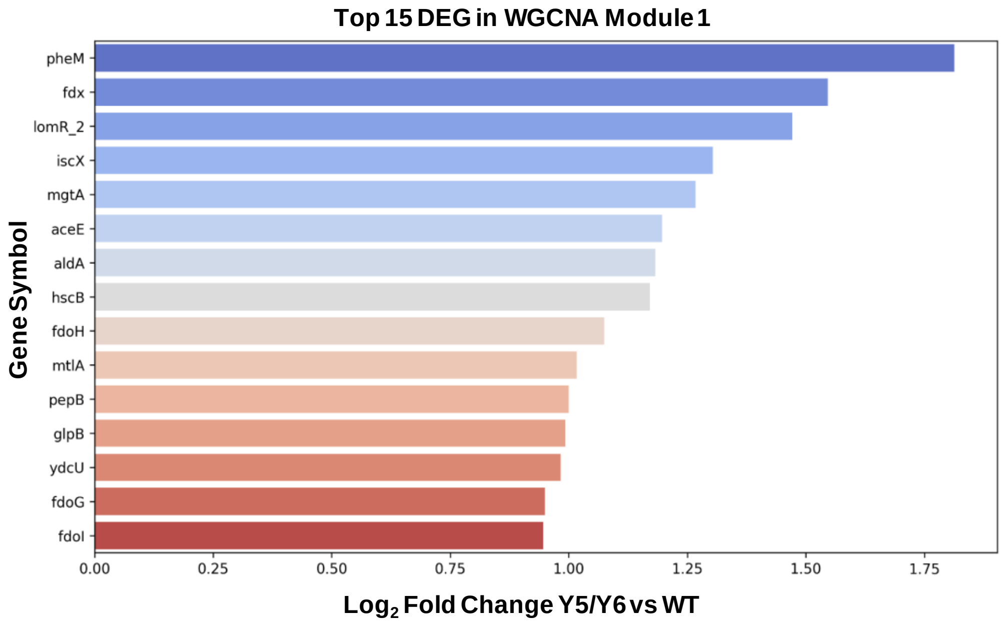

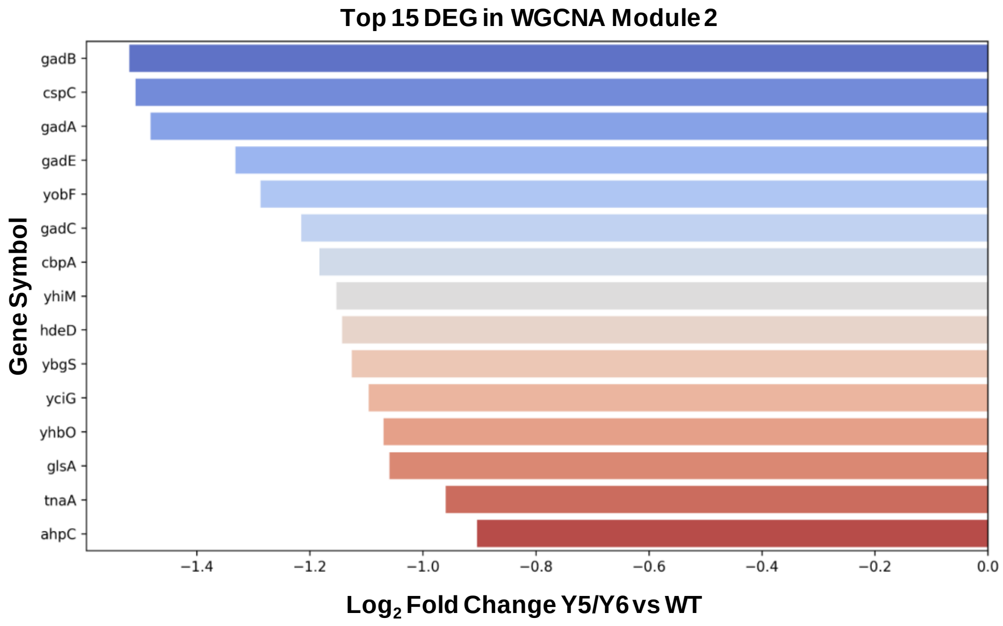

Supplementary Figure S7. Protein-protein interaction (PPI) network of Module 1 genes (turquoise)

Protein-protein interaction (PPI) network of genes from Module 1 (turquoise).

Genes belonging to WGCNA Module 1 (turquoise) were filtered for high module connectivity ($kME_{turquoise} > 0.3$) and analyzed using STRING v12 for *E. coli* MG1655.

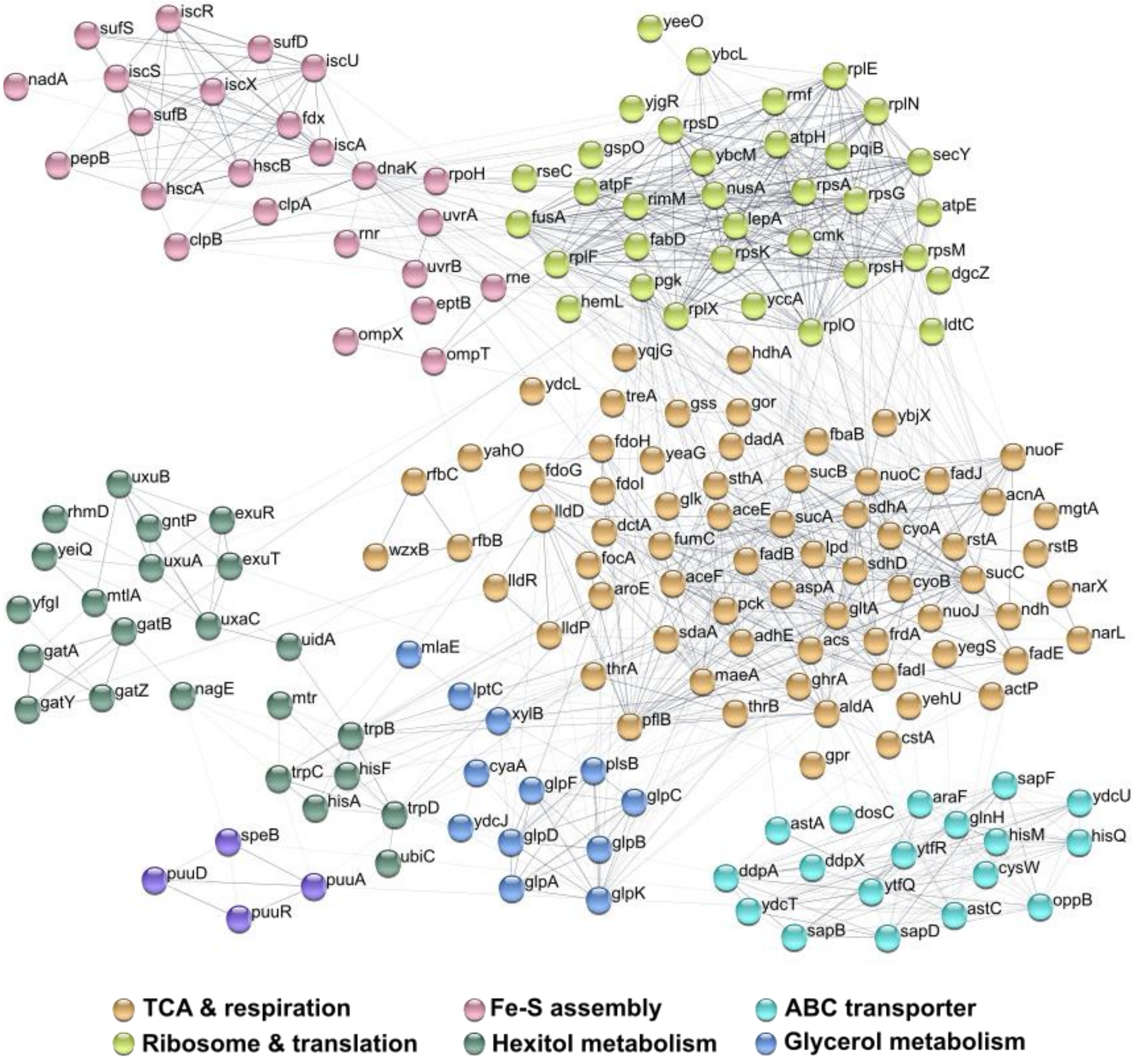

The network is displayed as a confidence-weighted interaction graph in Cytoscape. Nodes are colored according to unsupervised k-means functional clustering as defined by STRING. Each color represents a group of proteins sharing related biological functions (e.g., translation, energy metabolism, Fe-S cluster assembly, or stress response). Edge thickness

reflects the confidence of predicted interactions. Only clusters containing more than three proteins were retained for clarity.

Supplementary Figure S8. Protein-protein interaction (PPI) network of Module 1 hub genes

PPI network of the 44 most highly connected genes (“hub genes”) from Module 1 (turquoise), defined by WGCNA based on strong module membership (kMEturquoise > 0.95).

The network was generated using STRING (v12.0) for *E. coli* MG1655 and visualized in Cytoscape.

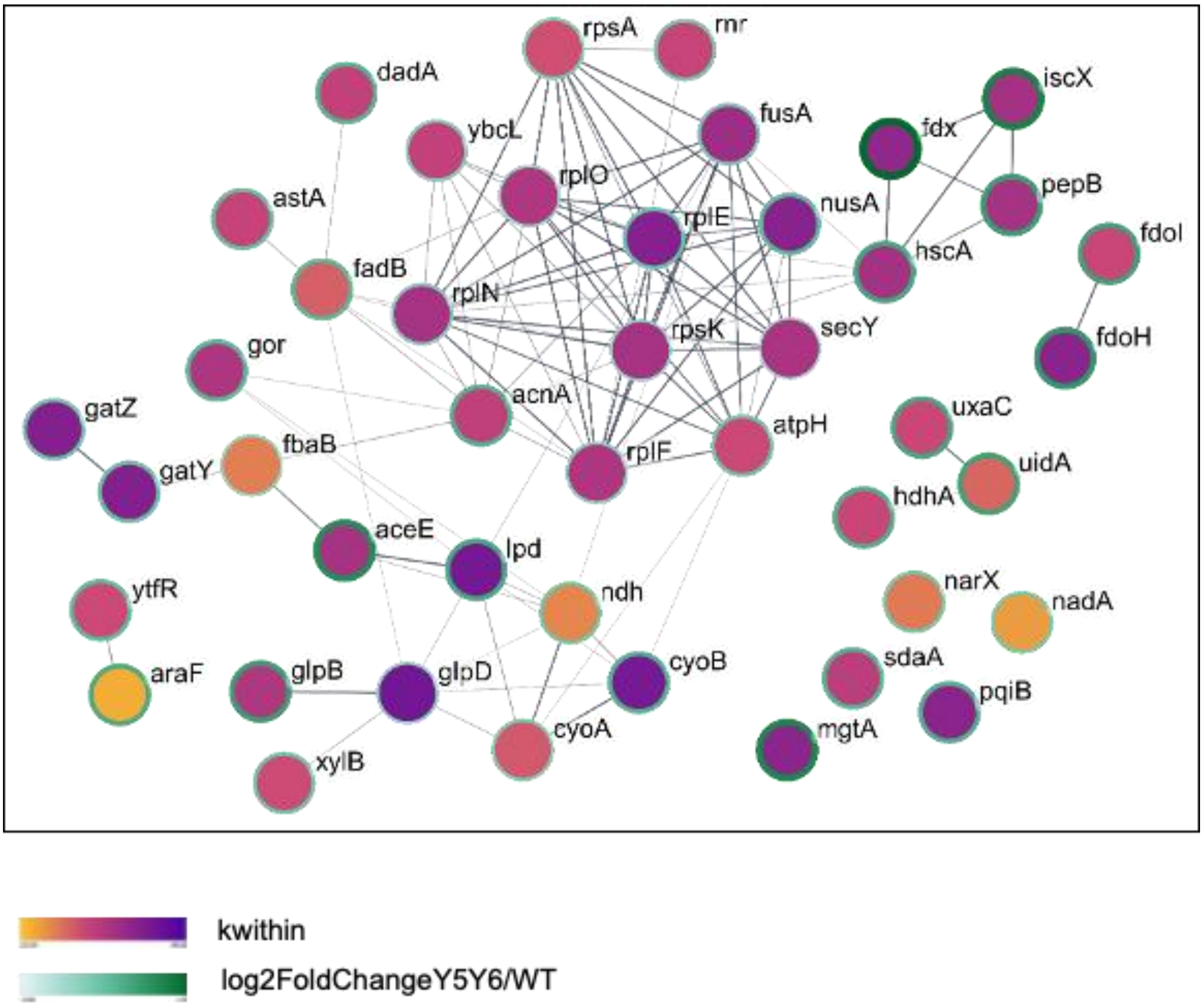

Node fill color represents the strength of intra-module connectivity (kWithin), with darker shades indicating more central “hub” positions within the co-expression module.

Node border color and width reflect the log_2-transformed fold change in gene expression between Y5/Y6 and WT strains, highlighting differentially expressed genes (all these genes are upregulated in Y5/Y6 compared to WT).

Only high-confidence protein–protein interactions are shown (STRING score > 0.7).

Supplementary Figure S9. Protein–protein interaction (PPI) network of Module 2 genes (blue)

The network was constructed using STRING v12 for *E. coli* MG1655, and visualized as a confidence-weighted interaction graph. Nodes are colored according to unsupervised k-means functional clustering as proposed by STRING.

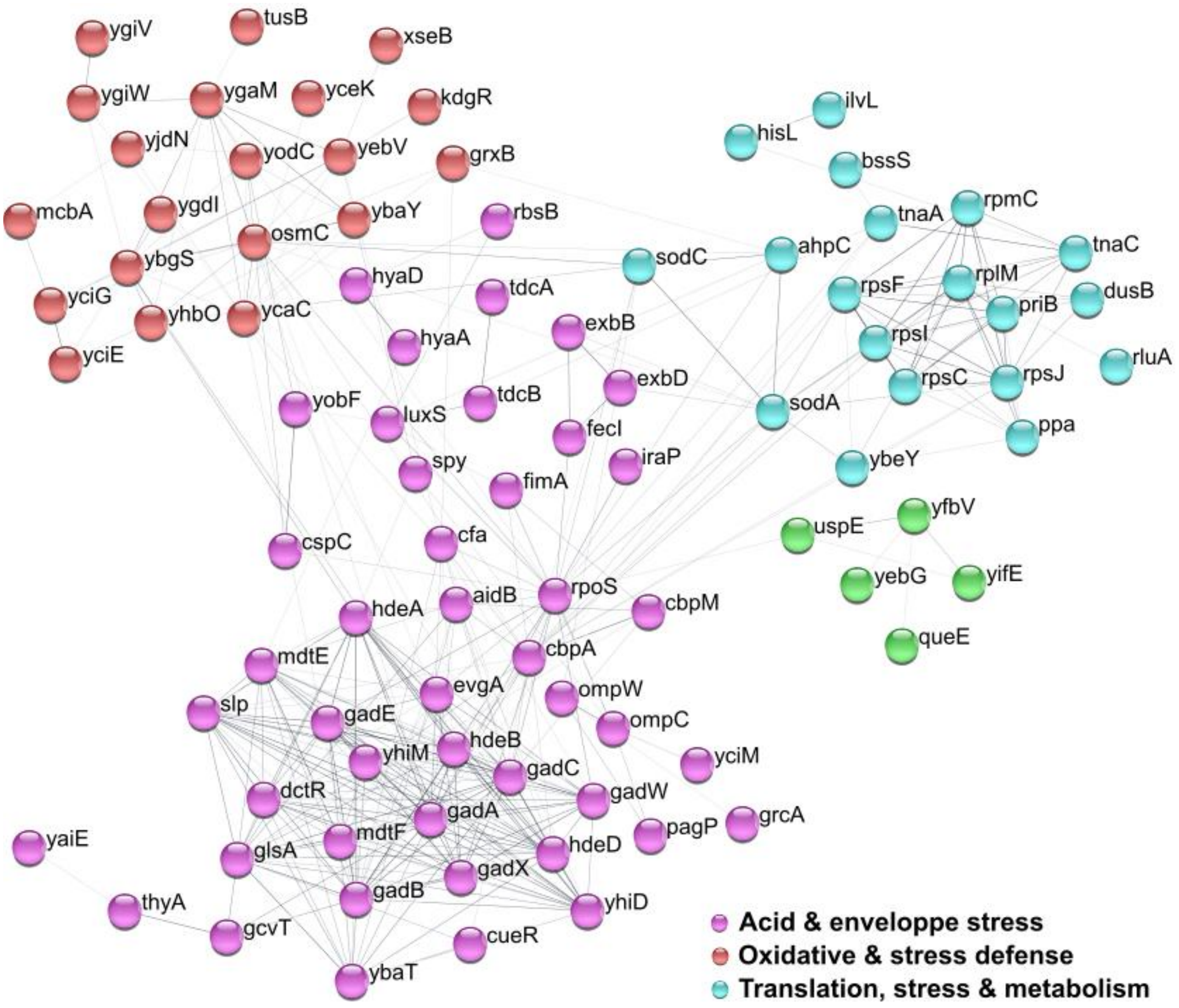

Each color highlights a group of proteins sharing related biological functions (e.g., translation, energy metabolism, stress response). Main cluster categories were simplified and annotated

manually for interpretability in the figure. Edge thickness represents interaction strength. Only clusters containing more than three proteins were retained for clarity.
