# Supplementary material for: Genomic diversification, adaptive convergence, and regulatory rewiring in aging Escherichia coli colonies
Source: BMC Microbiol. 2026 Mar 31;26:455. doi: 10.1186/s12866-026-04983-z (PMC13162491; doi:10.1186/s12866-026-04983-z)
Supplement: Supplementary file 5 — Additional file 5: Unprocessed PCR gel images for Supplementary Figure S2B. [file 12866_2026_4983_MOESM5_ESM.pdf]

Manuscript: Genomic diversification, adaptive convergence, and regulatory rewiring in aging Escherichia coli colonies

Authors: Claude Saint-Ruf, Adrien Launay, Olivier Tenaillon, Ivan Matic

Additional file 5: Unprocessed PCR gel images for Supplementary Figure S2B

Supplementary note (PCR gels). Gel 1 provides the full-length, original unprocessed PCR gel corresponding to the cropped panel shown in Supplementary Figure S2B (with an annotated version indicating lanes and the cropped region). Gels 2 and 3 provide full-lane context for PCR screening of the *yobF-cspC* locus across isolates (Y1-Y14 and Y15-Y25) and include WT and no-template controls; Gel 2 provides an independent PCR replicate for strains Y5 and Y6 shown in Supplementary Figure S2B. PCR amplification of the *yobF-cspC* locus was performed using primers PyobFcspC1 and PyobFcspC2 (WT expected amplicon: 1.8 kb); PCR products were subsequently confirmed by Sanger sequencing.

Gel 1 (raw): Uncropped, unprocessed original image corresponding to Supplementary Figure S2

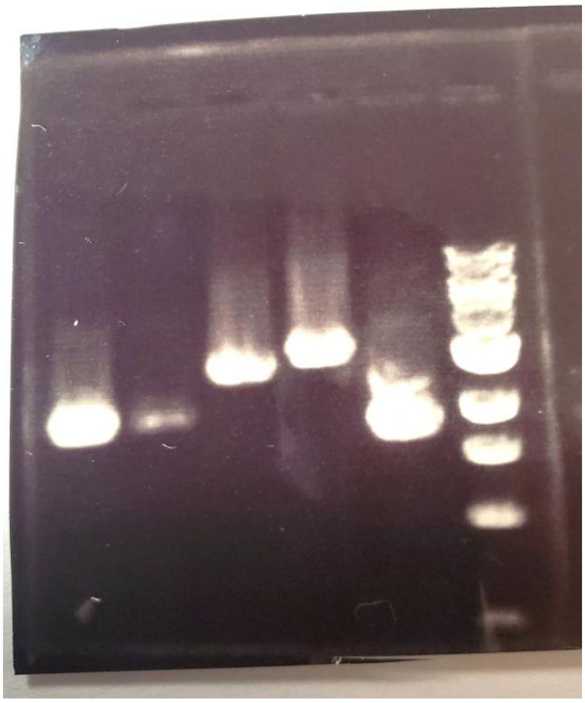

Gel 1 (raw). This is the agarose gel used for the cropped PCR panel in Supplementary Figure S2B. The full agarose gel is shown with all edges visible. No cropping or local image adjustments were applied to this image.

Gel 1 (annotated): Lane identification and region cropped for Supplementary Figure S2

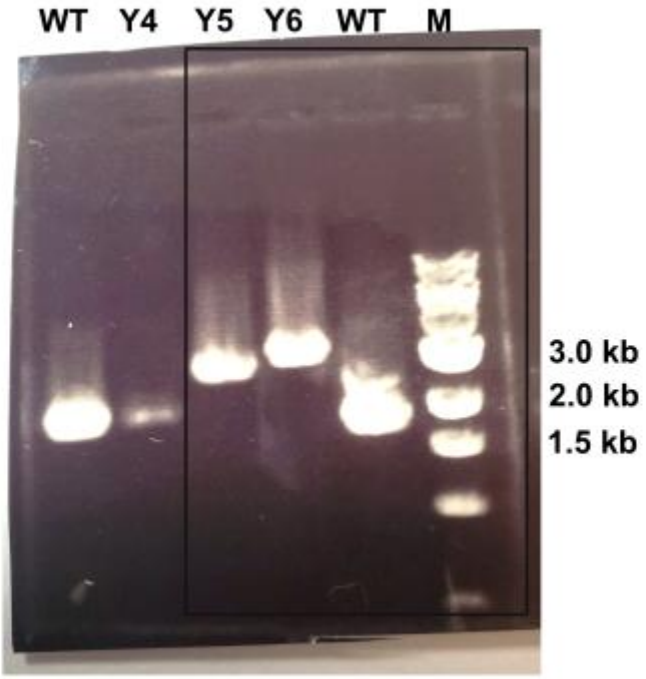

Gel 1 (annotated). Annotated version of the original gel shown in Page 1. Lanes are labelled (WT, Y4, Y5, Y6, WT) and M indicates the DNA ladder. The boxed area indicates the region that was cropped and shown in **Supplementary Figure S2**. The underlying gel image is identical to the raw image (Page 1); annotations are overlays only.

Gel 2+ Gel 3 (screening)

Gel 2 : PCR products for strains Y1-Y14 and controls

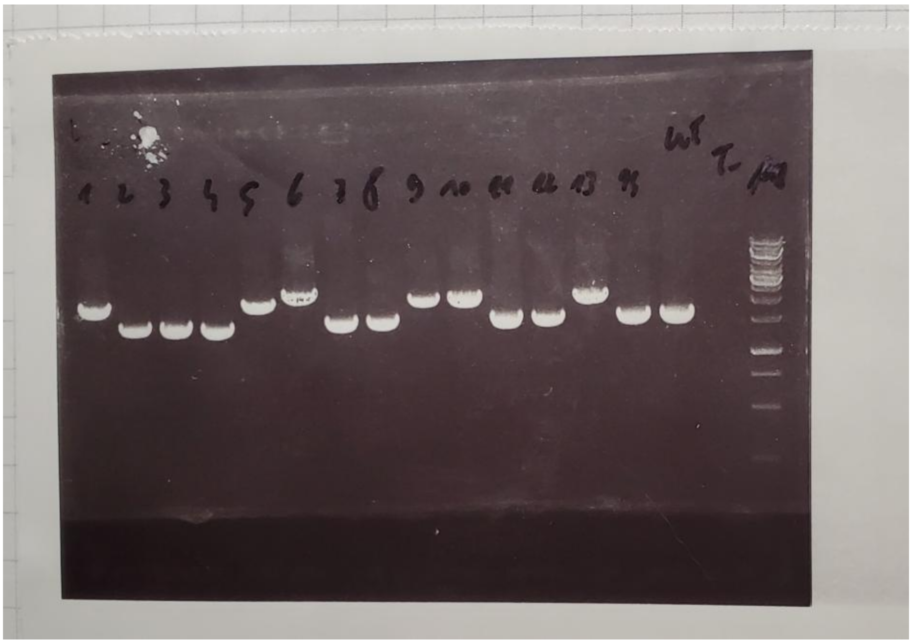

Gel 2 lane key: Lane 1: Y1; Lane 2: Y2; Lane 3: Y3; Lane 4: Y4; Lane 5: Y5; Lane 6: Y6; Lane 7: Y7; Lane 8: Y8; Lane 9: Y9; Lane 10: Y10; Lane 11: Y11; Lane 12: Y12; Lane 13: Y13; Lane 14: Y14; Lane 15: WT; Lane 16: water (no-template control); Lane 17: M (1 kb DNA ladder)

Gel 3: PCR products for strains Y15-Y25 and controls

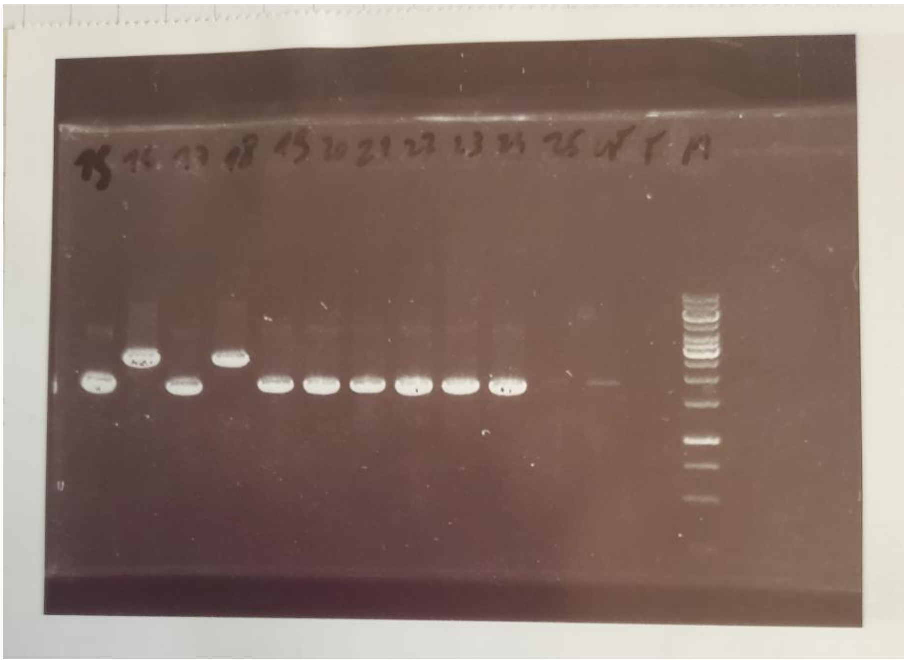

Gel 3 lane key: Lane 1: Y15; Lane 2: Y16; Lane 3: Y17; Lane 4: Y18; Lane 5: Y19; Lane 6: Y20; Lane 7: Y21; Lane 8: Y22; Lane 9: Y23; Lane 10: Y24 (not included in this study due to

unsuccessful genome sequencing); Lane 11: Y25; Lane 12: WT; Lane 13: water (no-template control); Lane 14: M (1 kb DNA ladder).

Gels 2-3 (additional screening). Full-length gel images showing PCR products for screening of the *yobF-cspC* locus across evolved isolates, with WT and no-template (water) controls and M (1 kb DNA ladder). Gel 2 includes strains Y1-Y14 and Gel 3 includes strains Y15-Y25. As expected, isolates carrying an insertion within the locus yield larger PCR amplicons than WT; in these gels, larger amplicons are observed for Y1, Y5, Y6, Y9, Y10, Y13 (Gel 2) and Y16, Y18 (Gel 3), consistent with WGS-based annotations. Gel 2 also serves as an independent replicate for the Y5 and Y6 PCR products shown in Gel 1. Note: in Gel 3, lane Y25 shows a weak PCR signal and the WT signal is faint; the archived photograph does not capture the right lateral edge of the gel.
